# Time-resolved chemical monitoring of whole plant roots with printed electrochemical sensors and machine learning

**DOI:** 10.1126/sciadv.adj6315

**Published:** 2024-01-31

**Authors:** Philip Coatsworth, Yasin Cotur, Atharv Naik, Tarek Asfour, Alex Silva-Pinto Collins, Selin Olenik, Zihao Zhou, Laura Gonzalez-Macia, Dai-Yin Chao, Tolga Bozkurt, Firat Güder

**Affiliations:** ^1^ Imperial College London, Department of Bioengineering, Royal School of Mines, SW7 2AZ London, UK.; ^2^ Shanghai Center for Plant Stress Biology, CAS Center for Excellence in Molecular Plant Sciences, Chinese Academy of Sciences, Shanghai 200032, China.; ^3^ Imperial College London, Department of Life Sciences, Royal School of Mines, SW7 2AZ London, UK.

## Abstract

Traditional single-point measurements fail to capture dynamic chemical responses of plants, which are complex, nonequilibrium biological systems. We report TETRIS (time-resolved electrochemical technology for plant root environment in situ chemical sensing), a real-time chemical phenotyping system for continuously monitoring chemical signals in the often-neglected plant root environment. TETRIS consisted of low-cost, highly scalable screen-printed electrochemical sensors for monitoring concentrations of salt, pH, and H_2_O_2_ in the root environment of whole plants, where multiplexing allowed for parallel sensing operation. TETRIS was used to measure ion uptake in tomato, kale, and rice and detected differences between nutrient and heavy metal ion uptake. Modulation of ion uptake with ion channel blocker LaCl_3_ was monitored by TETRIS and machine learning used to predict ion uptake. TETRIS has the potential to overcome the urgent “bottleneck” in high-throughput screening in producing high-yielding plant varieties with improved resistance against stress.

## Teaser

A real-time sensing platform was developed to provide insights into ion uptake and chemical responses in whole living plants.

## INTRODUCTION

The development of stress-resistant varieties of plants that can tolerate biotic (e.g., fungi, bacteria, and viruses) and abiotic (e.g., salinity, drought, heat, cold, and extreme pH) stresses, while still producing high yields, is crucial to maintaining and improving crop production. Conventional (including glasshouse- and field-based) phenotyping (assessment of, for example, physical form and visual appearance) to select for stress-tolerant species and varieties typically only results in a single yield measurement for a set condition or growth season (*1*). Plants under stress, however, produce chemical signals that often vary in concentration or composition over time (*2*). For the accelerated development of stress-resistant species, measuring the behavior of the plant in real time under stressful, but realistic, simulated environmental conditions is, therefore, of great interest in both applied and fundamental research.

Simulated growth conditions (e.g., laboratory or glasshouse) can be combined with analytical methods to quantify biometrics, improving the efficiency and accuracy of rapid and automated phenotyping. Among various analytical techniques, optical approaches have probably been the most widely used in the research community for continuous-time phenotyping of whole, living plants. Depending on the phenotype in question, different optical methods can be used, some of which produce signals with spatiotemporal fidelity. For example, infrared (IR) thermography, a method for noninvasively measuring IR heat signatures, has been used to monitor stomatal conductance [degree of stomatal opening used for estimating CO_2_ and H_2_O exchange (*3*)] under conditions of salt stress, allowing estimation of robustness against salt stress (*4*). Positron emission tomography, which spatially maps the radiation emitted by a compound, has been used to estimate resistance to salt stress by measuring the uptake and transport of ^22^Na radioisotope in salt-resistant and salt-sensitive plant species (*5*). The capabilities of optical phenotyping can be expanded through the use of genetically encoded and engineered materials that can be introduced into plants. These materials can be used to monitor chemical signals such as pH or Ca^2+^ via detection of fluorescence to study stress responses from stressors such as insect feeding (*6*, *7*). Despite being a powerful approach for continuous-time phenotyping of whole plants in terms of analytical performance, optical sensing methods often require expensive and specialized equipment that may not be accessible or available to all laboratories. Because of the cost and complexity of optical approaches, they are often difficult to scale for mass phenotyping at an industrial scale. Interference from plant tissues and external light sources may also affect monitoring (*8*). There is also a lack of techniques that use both unmodified plants and standard analytes: typically, plants are modified (for example, with engineered materials) or a nonstandard analyte is administered (for example, radioisotopes). This restricts the applicability of many optical techniques to specific species or a limited range of analytes.

Electrochemical sensors for continuous-time phenotyping of whole plants emerge as an alternative to optical methods (*9*, *10*). Insertable electrochemical sensors (sensors that are inserted into plant tissues, such as stems or leaves) are typically formed from either conductive electrodes or microfabricated field-effect transistors and have been used to measure the internal chemical environment of plant tissues. Insertable sensors can detect bursts of H_2_O_2_ resulting from pathogenic and ultraviolet light stresses in leaf tissue, enabling characterization of responses to these stressors (*11*, *12*). Insertable sensors are, however, invasive (though minimally) and can potentially generate unintentional stress responses, reducing the signal-to-noise ratio for the signal of interest (*12*). Noninvasive electrochemical sensors for continuous-time phenotyping of whole plants come in different forms, including devices placed externally on plants for measuring gaseous analytes, including tattoo-like sensors (*13*, *14*). Both wearable and insertable sensors, however, may be physically too large and heavy for use with fragile seedlings, thus limiting their use to mature plants (*9*).

Despite the critical importance of the roots as the main entrance of water and nutrients for the plant, application of noninvasive electrochemical sensors to the roots for real-time, whole plant phenotyping has been underexplored. Because of the importance of pH in the root microenvironment, Felle (*15*) used a glass microelectrode to continuously measure the apoplastic pH of the root cortex of *Zea mays* (corn) to investigate the impact of various ions and compounds on pH regulation in the roots. Using the same system, Felle *et al.* (*2*) studied pH signaling on both the root surface and in the leaf apoplast of *Hordeum vulgare* (barley) upon pathogenic inoculation of the roots, demonstrating time-dependent response of pH under pathogenic stress. Glass pH microelectrodes, although effective, are fragile instruments that could not be easily handled by nonexpert users. Pt black/carbon nanotube–based microelectrodes were developed by McLamore *et al.* (*16*) for the detection of phytohormone indole-3-acetic acid (IAA) in *Z. mays* roots. IAA, which regulates response to abiotic stress, is taken into roots by both diffusion and transporters, and the microsensor demonstrated that influx occurred at different amounts at different locations around the root. Influx also showed an oscillatory pattern over the scale of minutes, with different mutants producing responses with different amplitudes and time periods. Although this work illustrates the precision microelectrodes afford, they are less useful for revealing whole-plant behavior and share the same fragility limitations as the glass electrodes by Felle *et al.* (*2*, *15*). These systems are also not compatible with mass fabrication, and hence, continuous-time phenotyping has not been adopted by the wider community to parallelize and automate phenotyping. Optical sensing has been used for high-throughput monitoring, where *Arabidopsis thaliana* seedlings grown in gel media in multiwell plates can be combined with robotic movement platforms to enable image capture from above, to measure plant growth via plant surface area, or from the side, to measure root development (*17*, *18*). In the latter system, developed by Burrell *et al.*, rows of eight samples at a time could be moved and placed in different wells containing chemical treatments that diffuse into the samples, allowing treatments to be applied after germination. Electrochemical sensing can extend the capabilities for plant root monitoring if the many advantages of printed sensors are exploited. Screen-printing is already commonly used in high-throughput manufacturing and is compatible with conductive inks, facilitating the production of highly customizable sensor arrays. Ion-specific membranes, biological recognition elements, catalysts, and many other materials and methods can facilitate continuous monitoring of a large range of analytes by electrochemistry without requiring expensive analytical reagents for optical measurements and complex optical instruments (*9*, *19*–*23*).

**Fig. 1.**
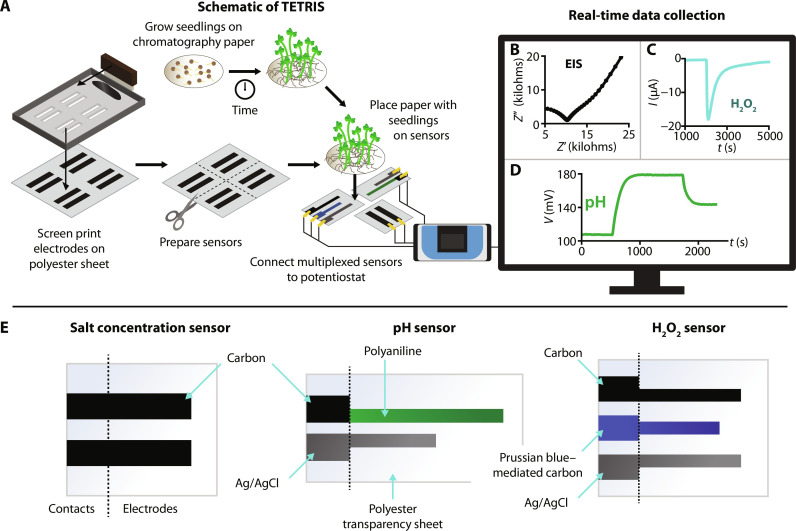
Setup of TETRIS. (**A**) Schematic of TETRIS, showing sensor fabrication, growth of seedlings, and recording of measurements using a standard laboratory potentiostat. (**B**) Electrochemical impedance spectroscopy Nyquist plot of a paper disc with 30 kale seedlings (9 days old). (**C**) H_2_O_2_ sensing during the addition of H_2_O_2_ (1 mM, 20 μl) to a paper disc with KCl (1 M, 180 μl). (**D**) pH sensing during the addition of H_2_SO_4_ and NaOH to deionized water. (**E**) Design and materials of our salt concentration, pH, and H_2_O_2_ sensors; illustrations not to scale.

In this work, we report our whole-plant monitoring platform: TETRIS (time-resolved electrochemical technology for plant root environment in situ chemical sensing) (Fig. 1). TETRIS consists of three screen-printed low-cost sensing modules for the multiplexed measurement of concentrations of ions [by electrochemical impedance spectroscopy (EIS)], H_2_O_2_, and pH. Changes in pH and H_2_O_2_ levels can coincide with wounding and biotic stresses, such as pathogen infection (*24*). Ions vary in size, charge, and relevance to plants, where some are key nutrients and others are toxic heavy metals, and so tracking their uptake can be indicative of salt sensitivity or tolerance (*25*, *26*). As the rate of uptake of ions depends on charge gradients, the simultaneous measurement of multiple analytes can elucidate the interactions between these different chemical species (*27*). We have extensively characterized TETRIS and investigated the characteristics of uptake of ions in whole *Brassica oleracea acephala* (dwarf green curled kale), *Solanum lycopersicum* (tomato), and *Oryza sativa* (rice) plants, all common crop plants, continuously and in real time. We studied the significance of the substituent ions and their properties on the rate of uptake in kale and investigated the effect of ion channel blockers on the uptake of ions. Last, using the library of data collected using TETRIS, we developed a machine learning model that can predict the rates of uptake of salts.

## RESULTS

### General experimental setup of TETRIS

To measure the chemical environment of the roots of plants, we developed a sensing platform, TETRIS, comprising a measurement chamber and disposable sensing module (movie S1). Sensors were created by screen-printing conductive ink onto plastic transparency sheets. Screen-printing is a technique that is low-cost, is used industrially, and does not require highly specialized equipment, meaning that transfer of these sensors from a laboratory to a production scale is reasonably straightforward. The sensors are then individually cut (and for the pH sensor, electrochemically treated) and attached to a raised platform.

**Fig. 2.**
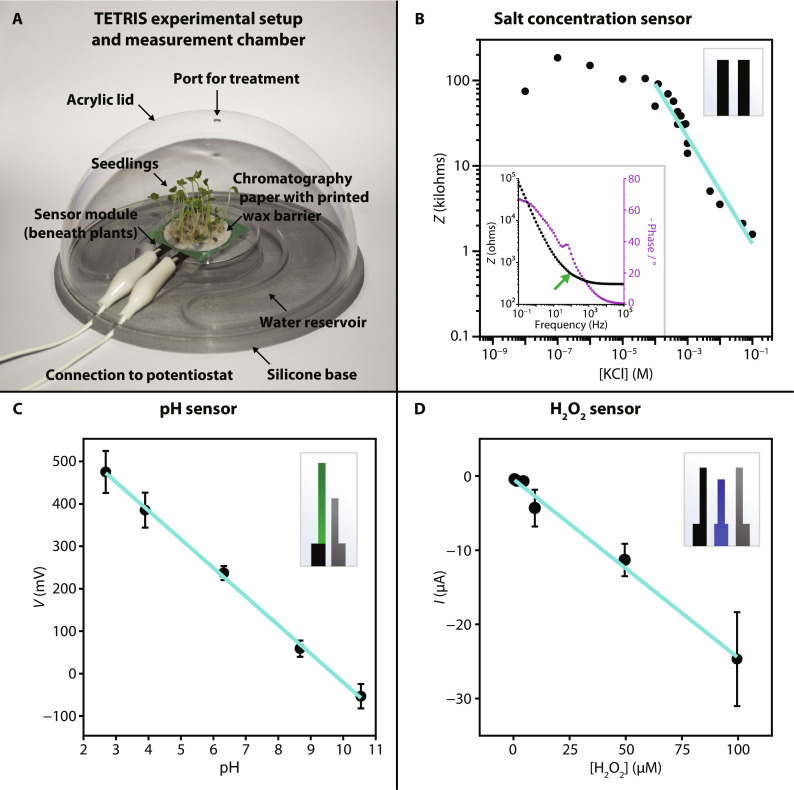
Characterization of TETRIS. (**A**) Photograph of TETRIS, where single or multiple sensors can be placed under seedlings grown on paper (kale seedlings pictured). (**B**) Average impedance response of our salt concentration sensor to various concentrations of KCl at 2 kHz. Solid teal line indicates linear fit of linear range of data. Inset shows the Bode plot of electrochemical impedance spectrum in 0.1 M KCl, where the green arrow shows our chosen frequency of 2 kHz. (**C**) Calibration curve of pH sensor in 1 M KCl. Error bars show 1 SD (*n* = 8). (**D**) Calibration curve of the H_2_O_2_ sensor in 1 M KCl. Error bars show 1 SD (100 μM: *n* = 4; 2 μM: *n* = 2; 5 to 50 μM: *n* = 3). Sensor design displayed in the top right corner of each plot (B to D).

The module itself can hold a single sensor or multiple sensors in either stacked or lateral positioning to form a multiplexed sensing system (Fig. 1A and fig. S1). The multiplexed nature of TETRIS allows simultaneous sensing of different analytes in the same sample, including concentrations of salts (via EIS), H_2_O_2_, and pH, where we chose these analytes due to their relevance as signals in plant physiological processes and stress response (Fig. 1, B to E) (*9*). The sensing module, holding a single sensor (or multiple sensors for multiplexed measurements), was placed into the chamber, consisting of a silicone base and transparent acrylic lid (Fig. 2A). A reservoir of water was created in the silicone base to maintain a constant humidity (see fig. S2). The sensors were connected to a standard laboratory potentiostat (PalmSens 4 by PalmSens BV, Netherlands) with two eight-channel multiplexers for multiplexed measurements to allow for up to 16 simultaneous recordings (fig. S1C). The potentiostat was connected to a desktop personal computer for real-time measurements.

All experiments were performed with the plants grown on discs of chromatography paper (Whatman 1). Seeds were first germinated on wet tissue and transferred onto chromatography paper, where hydrophobic wax barriers were created by a Xerox ColorQube solid wax printer to define the growth and measurement area of the paper disc. The presence of a wax barrier also prevented the analyte solution from reaching the contacts and shorting the measurement circuit. The plants were grown in enclosed boxes with a constant water supply for a set number of days, in batches large enough for each experiment. The paper substrate with the plant seedlings was then placed onto the sensor module to start the measurement.

We designed three sensors for use in TETRIS: (i) an impedance-based nonspecific salt concentration sensor, (ii) a potentiometric pH sensor, and (iii) an amperometric H_2_O_2_ sensor (Fig. 1, B to E, and fig. S3). Calibration curves for the sensors are shown in Fig. 2 (B to D), and the full characterization can be found in the Supplementary Text.

### Continuous measurement of chemical signals in the plant root environment

**Fig. 3.**
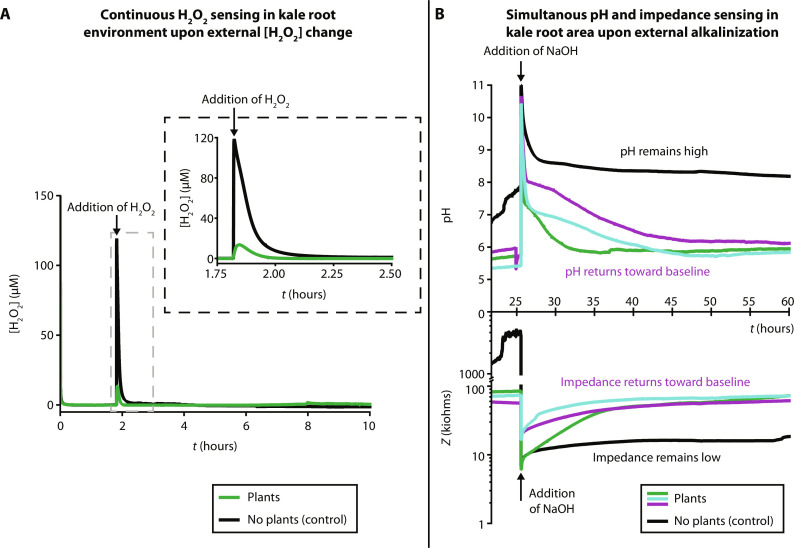
Real-time chemical monitoring of the plant root environment. (**A**) Real-time monitoring of H_2_O_2_ concentration in the root environment of kale seedlings (green signal, 10 plants, 16 days old) and blank paper control (black signal), with the addition of H_2_O_2_ (500 μM, 30 μl) into the paper disc (black arrow). Dashed border inset shows magnified plot. (**B**) Real-time, simultaneous monitoring of pH (top) and electrical impedance (bottom) of the root environment of kale seedlings (green, purple, and cyan signals; 30 plants; 9 days old) and blank paper control (black signal), with the addition of NaOH solution into the paper disc (black arrows). Data smoothed in OriginPro using a percentile filter (50th percentile, 30 points of window).

We used TETRIS to monitor in real time the chemical environment in the roots of plants grown on paper. After growing for a certain time, the paper disc with seedlings was removed from the growth box and placed onto the sensing module (Fig. 2A). Figure 3 shows the continuous measurement of impedance, pH, and H_2_O_2_ in this setup. Figure 3A displays the addition of H_2_O_2_ to the roots of kale plants, where the increase in signal upon addition is visible. The control (chromatography paper composed of cotton cellulose, with no seedlings) showed a larger peak than with kale seedlings upon the addition of H_2_O_2_ (30 μl, 500 μM) to the paper substrate. This difference in signal could be attributed to enzymes produced by the roots removing H_2_O_2_, where peroxidases are especially abundant in roots, and the physical presence of the roots slowing down diffusion (*28*). H_2_O_2_ has been administered externally to seedlings by a variety of methods (spraying on foliage, through nutrient solution) and in a range of concentrations (1 μM to 15 mM) as a method of priming plants for abiotic stresses (such as salt stress and extreme temperature) (*29*–*32*). TETRIS could be used for monitoring H_2_O_2_ levels upon such H_2_O_2_ treatment. We also attempted to measure H_2_O_2_ production as a result of mechanical wounding to the roots, but the results obtained were unclear as to whether any signals observed were noise from the physical manipulation or from a plant response (fig. S4). TETRIS may be unable to detect these small (<2 μM) H_2_O_2_ signals without improvement of sensitivity. Future work modifying the electrode surface (for example, by nanoparticle addition, as outlined in the “Characterization of sensors” section in the Supplementary Text) could enable TETRIS to detect bursts of reactive oxygen species (such as H_2_O_2_) released upon detection of pathogens by the plant, upon physical wounding or from abiotic stress, which lie in the submicromolar range (*23*, *33*–*35*).

We also simultaneously measured real-time impedance and pH changes, placing the two sensors adjacently, where our aim was to demonstrate the multiplexing capabilities of TETRIS (Fig. 3B). As the potentiostat multiplexer switched between each measurement channel in turn, there was no undesirable noise or electrical interference between sensors, even when using all three sensors simultaneously (fig. S5). We modulated the external pH by adding a basic solution of a different pH to observe any time-dependent effects on the pH and impedance of the sample. Initially, the kale samples had a lower impedance and pH than control samples (chromatography paper discs without kale seedlings) due to the presence of nutrients in the seeds and plant root exudates, including ions, amino acids, hormones, and other molecules (*36*–*39*). The pH of the samples was adjusted with NaOH solution (pH 9, 300 μl), performed to simulate saline-alkali stress (previously explored in the literature for studying the effects of damage to roots) and the pH value chosen to match the value of soil considered alkaline (*40*, *41*). A rise in pH and a lowering in impedance were initially observed for all samples. Over the course of the following 35 hours, however, we observed in the kale samples a lowering of pH below 6.5 and an increase in impedance back toward their respective initial baseline values. Meanwhile, the pH of the control sample remained above pH 8 for at least 30 hours after addition and the impedance remained below 20 kilohms. The increase in impedance observed in the kale samples was attributed to ion uptake into the plant by the roots. Plants have evolved a variety of mechanisms to take up nutrients through the roots, including primary active transport (adenosine triphosphate moves nutrients across membranes, going against the nutrient’s gradient), secondary active transport [H^+^–adenosine triphosphatase (ATPase) drives uptake through existing gradients by creating electrical and proton gradients], membrane-bound transport proteins, and passive diffusion (*42*). It is likely that the Na^+^ ions were taken up by the roots of the plants, and as charge balance must be maintained, the root cells release H^+^ ions (mainly through H^+^-ATPase) to maintain this balance (*43*). These extruded H^+^ ions may then neutralize OH^−^ ions located at the root surface, reducing the pH.

### The uptake of ions by kale, tomato, and rice seedlings

**Fig. 4.**
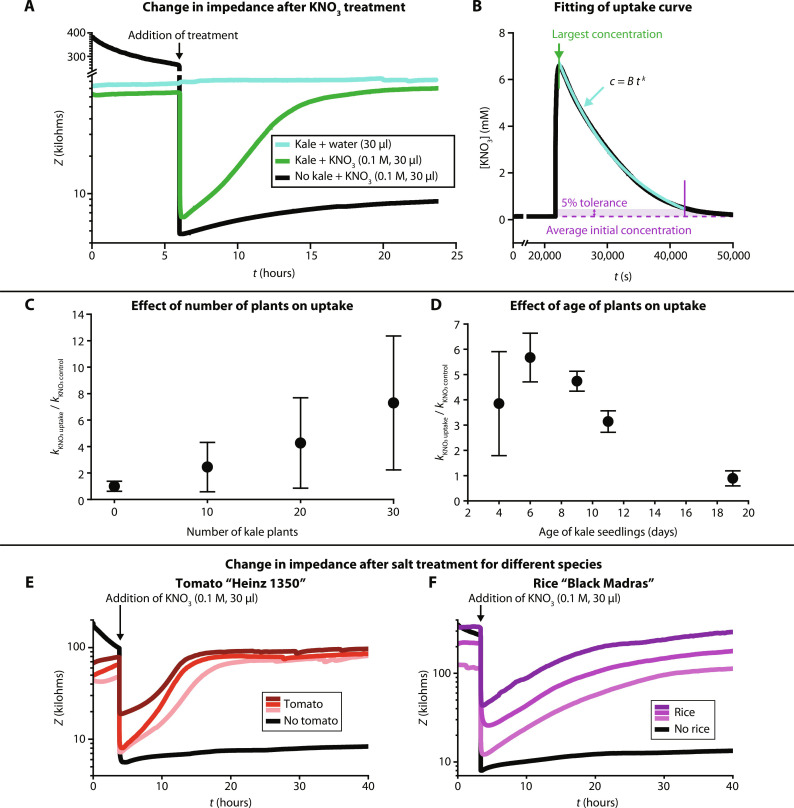
Uptake of ions by kale, tomato, and rice seedlings. (**A**) Kale seedlings grown on filter paper were placed onto the impedance sensor within TETRIS, and the electrical impedance was measured in real time. KNO_3_ (0.1 M, 30 μl) was then added to the filter paper after a rest period of at least 3 hours (arrow). The impedance slowly increased as ions were taken up by the seedlings and the salt concentration in the paper decreased (green signal). This is compared to empty paper substrate (no plants present), where the impedance remained low (black signal), and plants on paper with only water added instead of salt solution, where the impedance remained high (teal signal). Data smoothed in OriginPro using a percentile filter (50th percentile, 100 points of window). (**B**) An exponential curve of the form *c* = *Bt^k^* was plotted between the point of largest salt concentration measured up to where the curve meets the initial baseline concentration, with 5% tolerance. *k* was found and set as the uptake amount. (**C**) Effect of number of 9-day-old kale seedlings on relative amount of uptake of constituent ions of KNO_3_. Error bars indicate 1 SD (*n* = 3). (**D**) Effect of age of kale (30 plants) on relative amount of uptake of constituent ions of KNO_3_. Error bars indicate 1 SD (*n* = 3). Adding salts to different species: (**E**) KNO_3_ (0.1 M, 30 μl) added to 20 tomato Heinz 1350 seedlings on paper (red signal) or empty paper substrate (black signal) and (**F**) KNO_3_ (0.1 M, 30 μl) added to eight rice Black Madras seedlings on paper (purple signal) or empty paper substrate (black signal). Data smoothed in OriginPro using a percentile filter (50th percentile, 100 points of window).

Next, we investigated the increase in impedance observed over time in Fig. 3B in more detail using TETRIS to understand the uptake dynamics of various ions from inorganic salts by kale, tomato, and rice seedlings (movie S2). A real-time impedance measurement with added KNO_3_ can be seen in Fig. 4A, where the measured impedance after adding salt solution to kale is compared to addition of water to kale and addition of salt to chromatography paper only (composed of cotton cellulose, without seedlings). After an initial period for stabilizing the response of the sensors, the addition of 30 μl of water onto the paper substrate containing the seedlings did not cause a notable change in impedance, whereas addition of KNO_3_ (0.1 M, 30 μl) led to a large drop in impedance; a drop of ~60 kilohms was observed for seedlings and a larger drop of ~260 kilohms was observed without seedlings due to the higher initial starting impedance. The impedance remained steadily low in the blank filter paper without any seedlings, but in the sample with kale seedlings, the impedance increased over time, reaching the initial impedance level of the sample, suggesting uptake of K^+^ and NO_3_^−^ ions.

We first produced calibration curves for the salt concentration sensors, which correlate the impedance readings to salt concentrations (table S1). To then determine the change in salt concentration due to uptake, concentration (*c*) was plotted against time (*t*), and a logarithmic curve was fitted of the form *c* = *Bt^k^* (where *B* is a constant) to find the exponential term *k*_uptake_, the rate of uptake of the constituent ions of the salt (Fig. 4B). A small increase in impedance over time (~0.1 kilohms hour^−1^; see black signal in Fig. 4A) was observed in all impedance measurement, possibly due to changes of the microstructure of the polymer electrodes as a result of wetting and adsorption of ions present in solution in and on the electrode (*44*). To make a true comparison between different salts, *k*_uptake_ was normalized to the increase in impedance due to evaporation (*k*_control_). *k*_control_ was found in the same way as *k*_uptake_ but where no plants were grown on the paper.

We characterized the rate of uptake of K^+^ and NO_3_^−^ (addition of 0.1 M KNO_3_, 30 μl) versus number of kale seedlings grown on the paper substrate as shown in Fig. 4C. With the increasing number of plants, the collectively measured rate of uptake also increased in a linear fashion, likely due to increased root surface area and therefore higher overall collective rates of transport. Although this experiment produced results that were expected, strong evidence was generated, confirming the functionality of TETRIS for measuring rates of uptake for dissolved ions. We also demonstrated uptake of ions by the roots via an electrolyte leakage assay (a widely used method in plant research) of the stems of the kale seedlings (fig. S6) (*45*, *46*).

Next, we studied the relationship between the rate of uptake of ions from the added salts and the age of plants used in the experiment (Fig. 4D). We observed that the rate of uptake of ions increased with the age of plants, up to plants aged 6 days (their age from germination until the start of the experiment), albeit with large variation that was likely due to the variation in root growth of the young plants. For plants aged between 6 days and 19 days, the rate of uptake of ions decreased with the age of the plants used in the experiment. At 19 days old, the rates of uptake with plants were not notably different from the paper control without plants. This was an unexpected finding; although the older plants were visibly larger than younger seedlings, the rates of uptake were lower. Although we do not know exactly why, we speculate that because we do not provide any nutrients to the seedlings, other than deionized water during growth, the seedlings may not be developing as normal. This notion may also be supported by the observation that after about 2 weeks of growth on paper, some wilting, discoloration, and decay were generally evident in the plants. This plant degradation could have occurred due to microscopic cell death or lignification of the root tissue (*47*). Furthermore, unlike soil, paper is not a three-dimensional substrate, and hence, it does not provide the three-dimensional mechanical matrix that presumably allows support for proper root development or plant growth (*48*). In any case, the underlying biology of this observation will need to be studied in future experiments. We also observed that the values for uptake of K^+^ and NO_3_^−^ in 30 kale seedlings of 9 days differed in the experiments shown in Fig. 4 (C and D); this difference in uptake can be attributed to the seeds coming from different packs from the same manufacturer purchased over a year apart, where differences in environmental conditions of the mother plant can affect the quality of the seeds, including seed size and germination, a factor that was beyond our control (*49*).

Although seedlings at such a young stage generally do not require extra nutrients (due to the nutrients provided from the seed), we did explore in detail the compatibility of TETRIS with plants grown in a nutrient-rich solution, as many plant experiments in the literature are carried out with nutrients in the growth medium (*17*, *39*). We found that after growing plants in a multinutrient solution (composition listed in table S2), TETRIS was able to detect subsequent addition of KNO_3_ to the plants, despite the higher overall initial ion concentration (fig. S7). Similar values of *k*_uptake_/*k*_control_ were obtained for the addition of KNO_3_ to 30 kale seedlings (9 days old) grown in 1 and 10 mM Miracle-Gro nutrient solution (average values of 6.8 and 6.5 respectively, similar to those shown in Fig. 4C), suggesting that nutrient solution does not interfere with TETRIS. We also observed that TETRIS could monitor uptake of the multinutrient solution when added to the plants as with our other salt experiments. We decided to grow plants in deionized water for most of the experiments, however, to properly separate uptake of added nutrients and existing nutrients.

To demonstrate the nonspecificity of TETRIS to plant species, we briefly explored the uptake of ions by plants from different families (Fig. 4, E and F). Both tomato “Heinz 1350” and rice “Black Madras” were grown in a similar way to kale on discs of chromatography paper and transferred to the TETRIS measurement chamber, where impedance was measured in real time. After initial stabilization periods with each, KNO_3_ (0.1 M, 30 μl) was added to the tomato and rice, along with controls (with no plants). Both species displayed the same behavior as with the kale experiments, showing increase in impedance over time, attributed to the uptake of ions from the added salts. As each experiment used different numbers of seedlings and different growth times, direct comparisons between species cannot be made, but a similar behavior of the system suggests that TETRIS could be compatible with many plant species.

We also performed a preliminary experiment in agar, often used as a growth medium for plants, especially for *A. thaliana*, a model plant commonly used for plant research in the literature (*17*). We found that we were able to detect the uptake of K^+^ and NO_3_^−^ when the salt was added to the agar medium with kale plants, in a similar manner to our paper-based experiments (fig. S8). We did observe a great increase in impedance in most samples after 20 hours due to substantial evaporation of water from the agar medium. While conditions would need to be improved to prevent this evaporation, we believe that these initial experiments demonstrate the compatibility of TETRIS with conventional plant growth media, especially those already used frequently by plant scientists and that can support different plant species.

### Comparing the rates of uptake of nutrients, heavy metals, and sodium salts

**Fig. 5.**
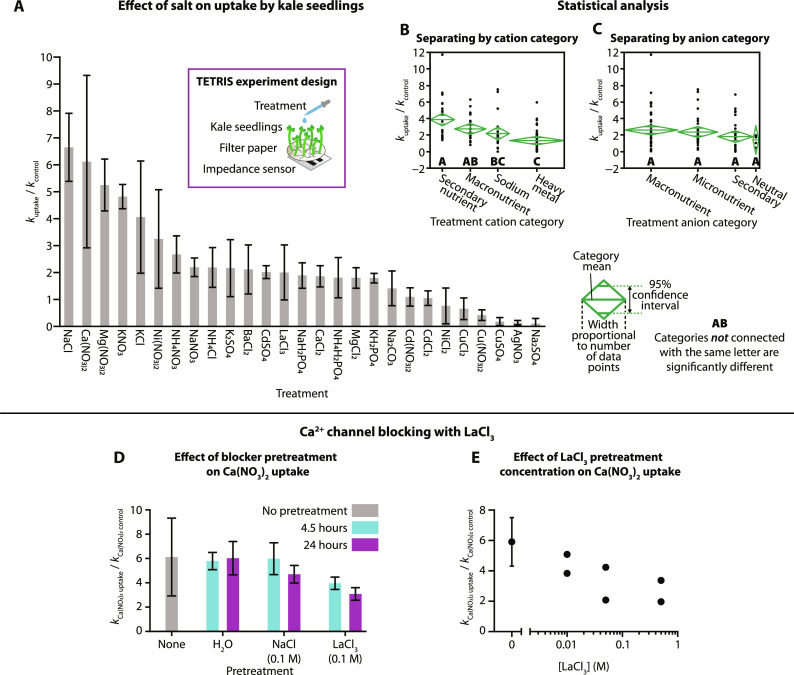
Effect of different salts on normalized rate of uptake. (**A**) Rate of uptake of salt from paper disc with 30 kale seedlings (9 days old), relative to control without plants, using TETRIS. (Values at or below 1 are considered to have a net zero or negative ion uptake.) Error bars indicate 1 SD [CdCl_2_, KCl, and NaCl: *n* = 3; CaCl_2_ and Ca(NO_3_)_2_: *n* = 6; LaCl_3_: *n* = 7; all others: *n* = 4]. Statistical analysis showed significant differences between some cation classes (**B**), but not between anion classes (**C**), where confidence diamonds show mean uptake, 95% confidence interval and number of data points, and letters show categories with or without significant difference. (**D**) Pretreatment of 30 kale seedlings (9 days old) with deionized water, NaCl (0.1 M), or LaCl_3_ (0.1 M) for 4.5 or 24 hours and their effect on the relative change in concentration of Ca(NO_3_)_2_. Error bars indicate 1 SD (none: *n* = 6; LaCl_2_ 24 hours: *n* = 4; all others: *n* = 3). (**E**) Pretreatment of 30 kale seedlings (9 days old) with varying concentrations of LaCl_3_ for 4 hours and the effect on the relative change in concentration of Ca(NO_3_)_2_. Error bars indicate 1 SD (*n* = 2).

To characterize the rates of uptake of different ionic species, kale seedlings were treated with salts containing a range of cations and anions to observe the effect. The change in salt concentration in paper discs with 30 kale seedlings was determined using TETRIS for each salt and compared to the recorded change in concentration for plain paper discs with no plants. Plants aged 9 days were selected due to their high rates of uptake without large deviation between samples (Fig. 4D). Normalized rates of uptake were more reliable than individual rates as processes such as evaporation caused a slight increase in impedance over time, as outlined before. A clear difference in normalized rate of uptake was observed between chemical species (Fig. 5A), where one-way analysis of variance (ANOVA) (*F*_26,88_ = 11.0164, *P* < 0.0001) showed statistical differences in uptake between many of the salts (fig. S9 and table S3).

To understand these observed differences in uptake, we compared the composition of the salts. Salts with ions considered to be nutritious for plants (macronutrients: K^+^ and NH_4_^+^; secondary nutrients: Ca^2+^ and Mg^2+^) or those with smaller cations (Na^+^) generally exhibited higher rates of uptake than those salts with heavy metal (HM) cations (Ag^+^, Ba^2+^, Cd^2+^, Cu^2+^, La^3+^, and Ni^2+^). This difference was also observed in our electrolyte leakage assay, where stem samples taken from plants treated with NaCl showed greater conductivity than those treated with Cu(NO_3_)_2_ or deionized water (fig. S6). Some salts showed large variance in uptake, likely due to the heterogeneity of whole living plants, and we observed some salts that went against the general trends of uptake [for example, Ni(NO_3_)_2_, a heavy metal salt, showed greater uptake of its constituent ions than MgCl_2_, a secondary nutrient]. By separating the ions into those categories (macronutrients, secondary nutrients, sodium, and HMs), we found statistical significance in the average rate of uptake according to ion category by one-way ANOVA (*F*_3,111_ = 10.9353, *P* < 0.0001). A Tukey-Kramer post hoc test (selected because of the uneven number of samples in each category) revealed significant pairwise differences between some of the categories, including between both macro and secondary nutrients and HMs, and the confidence intervals and means are displayed in Fig. 5 (B and C). While secondary nutrients had a greater average rate of uptake than macronutrients, this difference was not found to be statistically significant.

The differences in rate of uptake can be explained through the different methods of uptake and transportation of ions in plants. K^+^ is a macronutrient for plants (nutrients that are required and consumed in large quantities). K^+^ is taken up by multiple mechanisms, grouped into seven families of K^+^-permeable cation transporters, although not all of these transporters are exclusive to K^+^ (*50*). Na^+^, a toxic ion for plants, competes with K^+^ uptake through high-affinity potassium transporters and nonselective cation channels due to their similar chemical properties and ion sizes, explaining the high rate of uptake observed of Na^+^ salts (*51*). NH_4_^+^ is a common source of nitrogen for plants (alongside NO_3_^−^); high rates of uptake we observed could be attributed to NH_4_^+^ transport systems in the root plasma membrane, despite NH_4_^+^ concentrations rarely naturally exceeding 50 μM in soil (*52*). Ca^2+^ is another important nutrient for plants, with multiple transport mechanisms, including Ca^2+^-permeable ion channels, Ca^2+^-ATPases, and Ca^2+^/H^+^ exchangers (*53*). Ca(NO_3_)_2_ showed a relatively high rate of uptake for its constituent ions, although CaCl_2_ showed a much slower uptake. This is potentially due to Cl^−^, considered a micronutrient, being taken up less than the macronutrient NO_3_^−^. A similar relationship was also observed with salts of Mg^2+^, where the constituent ions of Mg(NO_3_)_2_ showed a relatively high rate of uptake compared to MgCl_2_. Similar to Ca^2+^, Mg^2+^ is a key nutrient, with specific transporters for uptake via the roots, and so we observed similar rates of uptake to Ca^2+^ (*54*).

Many HM ions are encountered by plants through human-derived industrial and agricultural activities. Excessive HM uptake can be detrimental to the health of the plant through oxidative stress, damage to cell structures, and substitution of nutrients at cation exchange sites (*55*). Consumption of crops containing toxic HMs by humans can lead to health problems through accumulation of HMs in the body, indicating the importance of investigating plant uptake of HM ions. Furthermore, specific plants are often used to remove HMs from contaminated soil through “phytoremediation,” and therefore, plants that exhibit high rates of uptake of HMs are just as important as those that do not (*56*).

The rate of uptake would be expected to be low for most HMs due to a lack of transporters for nonessential HM ions, as was generally observed in our experiments (Fig. 5). HM ions, depending on their species, may be considered micronutrients (for example, Cu^2+^ is necessary for photosynthesis and Ni^2+^ activates urease activity) or potentially toxic pollutants, or sometimes either depending on the concentration. Essential HM ions may have specific transporters, but nonspecific diffusion-based uptake can occur for most HM ions, depending on the surrounding concentration. Ag(NO_3_)_2_ as Ag^2+^ and NO_3_^−^ was found to have a negligent amount of uptake, potentially due to its reduction to metallic silver, silver oxide, or AgCl, as suggested by the appearance of a gray discoloration forming on the roots of the plants (fig. S10) (*57*). Na_2_SO_4_ as Na^+^ and SO_4_^−^ also displayed negligible uptake, an unexpected observation as Na^+^ and SO_4_^2−^ (as a source of S) both typically displayed high rates of uptake. A previous study found that transport rates of Na^+^ are higher in treatments containing Cl^−^ than SO_4_^2−^, corroborating the lower rate of uptake for the constituent ions of Na_2_SO_4_ compared to that for NaCl, but such a low rate for Na_2_SO_4_ was unexpected (*58*, *59*). Cd^2+^ salts generally also had an observed low rate of uptake. It has previously been reported that cadmium ions are absorbed on the surface of the root but not beyond, suggesting that a minimal amount of cadmium is taken up by plants, in agreement with our observations (*57*).

Whereas separating the salts into their cation’s relationship to plants revealed statistical significance in rate of uptake, we did not find a similar significance when separating the salts by their anions. All the anions used here are considered either macronutrients (NO_3_^−^ and PO_4_^3−^), secondary nutrients (SO_4_^2−^), micronutrients (Cl^−^), or neutral (CO_3_^2−^). None of these anions are especially dangerous to plants (not even Cl^−^, which recently has even had a reevaluation as a possible macronutrient) and would only be considered toxic in large concentrations (4 to 35 mg g^−1^ dry weight) (*58*, *60*–*62*). Anions are also typically absorbed in excess compared to cations, thus are unlikely to be the limiting factor in uptake of salts as a whole of their constituent ions, and so, it is perhaps expected that we did not observe significant differences in uptake due to anion (*43*).

### Ca^2+^ channel blocking by LaCl_3_

The blocking of cation channels is of great interest for scientists looking at stress tolerances and reactions in plants. For example, internal processes (such as the result of wound response via effects of inducers, such as systemin, on Ca^2+^ signaling) and external processes (such as salt or nutrient stress via Na^+^ and nutrient uptake by root cells) can both be probed by using ion channel blockers (*63*, *64*). Certain HM ions can block the ion channels of smaller ions; for example, Ba^2+^ from BaCl_2_ has been shown by radiotracer experiments to decrease sodium uptake, and Ca^2+^ channels can be blocked by La^3+^ from LaCl_3_.

We investigated the La^3+^ blocking effect using TETRIS by pretreating plants with a blocker or a control before monitoring salt uptake. The paper and seedlings were then washed in deionized water to remove excess blocker and the plants were transferred onto fresh, prewetted paper discs. The impedance measurement was then initiated and, after a rest period of 24 hours, a 30-μl solution of 0.1 M Ca(NO_3_)_2_ was added. Initially, the effect of pretreating plants with LaCl_3_ (0.1 M), a known Ca^2+^ channel blocker, was compared to the effect of pretreating plants with NaCl (0.1 M), a salt not considered to block Ca^2+^ channels, and deionized water (Fig. 5D) (*64*). Compared to the normalized rate of uptake for Ca^2+^ and NO_3_^−^ without treatment (6.1 normalized uptake), we found similar rates of Ca^2+^ and NO_3_^−^ uptake when pretreating the plants for 4.5 hours with deionized water (5.8) or NaCl (6.0). Pretreating plants with LaCl_3_, however, led to a reduced rate of uptake for Ca(NO_3_)_2_ of around 4.0 (about a third reduction in uptake compared to the control). Increasing this pretreatment time from 4.5 to 24 hours, we found that a deionized water treatment produced a similarly high normalized rate of uptake for Ca^2+^ and NO_3_^−^ (6.0), but the addition of NaCl or LaCl_3_ did not. Treatment with NaCl led to a reduced rate of uptake of around 4.7 (a reduction of about a fifth), suggesting that NaCl may provide some blocking effect with longer treatment. It is also possible that excess NaCl remains on the surface of the roots between transfer to fresh paper disc. In any case, the blocking effect of La^3+^ on uptake of Ca^2+^ and NO_3_^-^ was still far more pronounced; increasing LaCl_3_ pretreatment time to 24 hours reduced the normalized rate of uptake of Ca^2+^ and NO_3_^−^ to 3.1, around half the uptake of the control.

Using TETRIS, we also investigated the effect of the concentration of LaCl_3_ on blocking Ca^2+^ channels. We prepared solutions of LaCl_3_ with four different concentrations ranging from 0 to 0.5 M. Next, the kale seedlings were pretreated for 4 hours with the LaCl_3_ solutions prepared before measuring the rate of uptake for Ca^2+^ and NO_3_^−^ using printed impedance sensors. We observed a marked decrease in the rate of uptake for Ca^2+^ and NO_3_^−^ with the increasing concentrations of LaCl_3_ (Fig. 5E) in a log-linear fashion. Although we did not go beyond 0.5 M, further increases in the concentration of the blocking agent LaCl_3_ or duration of pretreatment is likely to improve blocking of Ca^2+^ channels although clearly to a lesser extent.

The reduction of uptake of Ca^2+^ and NO_3_^−^ with La^3+^, but not complete prevention of uptake, suggests that cellular uptake may not be the only possible route of ion uptake. The seedlings also potentially absorb water, containing Ca^2+^ and NO_3_^−^, into xylem vessels. As the La^3+^ only blocks ion channels, uptake into the xylem from the roots via diffusion is still unhindered, and so some uptake of Ca^2+^ and NO_3_^−^ may have still occurred (*42*). With these results, we have shown the potential application of printed impedance sensors for studying continuous-time ion transport in plants, demonstrating the utility of printed impedance sensors in applied and fundamental plant research.

### Predicting rate of uptake of salts with machine learning

We developed a supervised machine learning model for TETRIS for predicting the rates of uptake for salts using only the physicochemical properties and the biological relevance of the constituent ions as inputs to the model to accelerate plant phenotyping. This approach may be most useful in identifying previously undiscovered regulators of ion uptake mechanisms and of cellular stress responses. This would subsequently lead to the development of new plant varieties with higher yields and higher tolerance to environmental fluctuations caused by climate change or soil salinization. Machine learning techniques (including neural networks and predictive clustering trees) have been used previously to estimate nutrient uptake of herbage in fields and water uptake in roots, but a modeling approach has not been used to monitor and predict uptake of ions from salts on the individual plant scale (*65*, *66*).

We used the eXtreme Gradient Boosting (XGBoost) algorithm for the development of the supervised machine learning model to predict the normalized rate of uptake (*k*_uptake_/*k*_control_). In simple terms, XGBoost is a scalable machine learning system for tree boosting—a method of combining many weak learners (or “trees”) into a strong classifier. The XGBoost method aims to find a relationship between the input *X* = {*x1*, *x2*, …, *xN*} and the output *Y* = {*y1*, *y2*, …, *yN*} (*67*, *68*). We built five models to predict *k*_uptake_/*k*_control_ within different ranges, from a simple two-range “uptake/no uptake” model to predicting *k*_uptake_/*k*_control_ with increasing resolution. We trained the model using the rates of uptake for the salts measured and physicochemical information on the individual ions in the salts, such as their chemical groups, relative masses, ion charges, and the biological relevance of the constituent ions to the plant (such as whether the ions were deemed to be nutrients)—see the Supplementary Text for the full list of features used in the model (*69*). The experimental dataset was split into five folds for cross-validation: in each validation, four folds (~80% of the data) were used for training and one fold, unseen data (~20% of the data), was used for testing, and cross-validation was carried out by changing which fold was used for testing. The classification models were evaluated using the *F*_1_ score (averaged over the different iterations of the cross-validation)—a metric for the accuracy of the model combining precision and recall, defined as

$$F_{1}=\left(2\cdot \text{precision}\cdot \text{recall}\right)/\left(\text{precision}+\text{recall}\right)$$

**Fig. 6.**
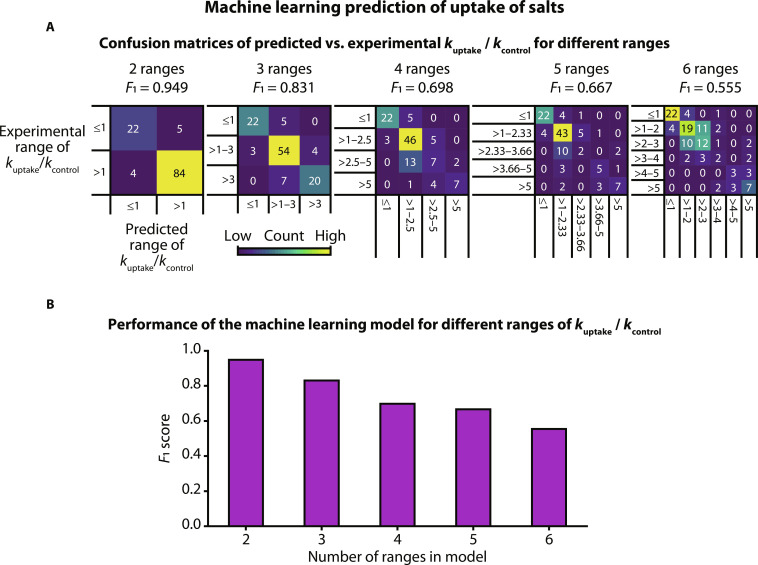
Using machine learning algorithms to predict normalized rate of uptake (*k*_uptake_/*k*_control_). (**A**) Confusion matrices comparing predicted and experimentally obtained ranges of (*k*_uptake_/*k*_control_). (**B**) *F*_1_ score (a metric of accuracy combing precision and recall) decreases with increasing number of ranges in the machine learning model.

We found that by using this machine learning approach, we were able to predict uptake with data from less than 100 experiments in the training datasets with reasonably high accuracy. Confusion matrices with five different range configurations can be seen in Fig. 6A. The two-range model, aiming to predict whether uptake did or did not occur (*k*_uptake_/*k*_control_ being >1 or ≤1, respectively), had an *F*_1_ score of 0.949, where “1” would be the perfect score for classification. As expected, increasing the number of ranges from two to six led to a decrease in *F*_1_ score (Fig. 6B) with six ranges producing the lowest score of 0.555.

## DISCUSSION

TETRIS is a low-cost platform for accelerated phenotyping for plants. As electrochemical root measurements are not used with living whole plants, TETRIS provides an important stepping-stone toward the measurement of physiological changes in whole plants, which often have a different behavior to individual plant organs or tissue samples. Seedlings can be challenging to measure with invasive electrical and electrochemical sensors because of the fragility of young plants. The roots are especially neglected in research, despite being the entry point for uptake of beneficial substances (water and nutrients) or harmful chemicals (sodium salts, heavy metals, and extreme pH). By measuring in real time, we not only enable information-rich observations into time-dependent chemical signals, but we unlock the potential for discovering previously unknown biology.

The entire TETRIS platform can be manufactured using tools, such as three-dimensional, wax, and screen printers, which are accessible to most academic laboratories. The total cost of materials to produce a single TETRIS system is US$5, excluding the multichannel potentiostat (which can be replaced with a lower cost commercial or a homemade alternative). Although we only performed experiment with three common crop plants (kale, tomato, and rice), the experimental setup was intended as a non–species-specific platform and is compatible with any seedlings that grow on paper or agar gel. *A. thaliana*, a member of the same family as kale (Brassicaceae), would be an ideal candidate for studying plant biology because of the wide range of mutants available, and the species is likely to be compatible with TETRIS. For example, using TETRIS with transporter mutants could elucidate the effects of altered transport mechanisms on the overall uptake of ions, such as nitrates (*70*).

The multiplexed nature of TETRIS allows us not only to measure multiple analytes at once but also to perform multiple experiments simultaneously, demonstrating its potential for use in high-throughput research (fig. S1). Where in our work we could run 16 sensors concurrently, the use of improved potentiostat technology and robotic automation could allow the simultaneous measurement of tens, hundreds, or even thousands of experiments at the same time, the scale that optical sensing can currently operate at (*17*).

TETRIS can also be used to examine the effects of environmental changes or stresses. For example, because of the boxed design, the internal atmosphere could be altered to increase or decrease the concentration of O_2_ or CO_2_, gases that are key for plant processes such as photosynthesis and respiration (*71*). The humidity could also be controlled by the size of the water reservoir or the lid, an environmental condition that has an effect on transpiration and photosynthesis (*72*).

There are five challenges for the TETRIS platform to overcome through future experimentation and technological advancement: (i) The electrochemical sensors must remain wet to function and are susceptible to drift if the water reservoir is depleted. (ii) The disposable sensors are printed on substrates made of polyester, which are not biodegradable and hence not environmentally friendly. The sensors, however, can be printed directly on paper to overcome this issue (*73*). (iii) In its current form, the TETRIS platform is not compatible with larger, mature plants; however, because of its scalable characteristic, the platform can be adapted to accommodate other model plants such as *Nicotiana benthamiana*. (iv) Measurement in soil may be less feasible due to the uneven geometry of soil; however, careful experimental design (such as carefully spreading of soil across the electrodes) may enable measurements in soil. (v) The relatively small number of experiments in the dataset (115 uptake experiments) limits the scalability of the machine learning models and has a higher potential for distortion due to outliers. Despite the current limitations, the machine learning approach we have developed could be used for the prediction of rates of uptake of various salts, such as those not previously tested. By including the concept of charge balance, machine learning could decouple uptake depending on the individual constituent ions and their interactions with each other as a salt.

Although, in this work, we mostly studied the rates of uptake of ions, with the existing TETRIS setup consisting of printed pH, EIS, and H_2_O_2_ sensors, biochemical signaling and feedback loops associated with, but not limited to, plant hormones, pesticides, and pathogenic or beneficial microorganisms can be studied (*9*). Because TETRIS is modular and customizable, the number of printed chemical sensors integrated into the system can be expanded to include sensors specific to certain ions or molecules (*20*). Combined, multiplexed measurement of both specific ions (e.g., Na^+^, K^+^, and Ca^2+^) and electrical impedance (not ion specific) could lead to the ability to decouple concentration of these ions from the impedance measurement, allowing the measurement of multiple ions simultaneously. Phytohormones, often the earliest markers for plant stress response, could be measured continuously and nondestructively using microneedle-based molecularly imprinted polymers (*74*). Aptamer-based sensors could be used to detect specific plant pathogens in the root environment (*75*). Heating and cooling elements in the silicone base could even be installed to control biorecognition element regeneration via temperature (*76*). Furthermore, TETRIS can be used as an artificial rhizosphere to study root microbiome to investigate plant-microbe interactions and potentially allow creations of digital twins of these complex systems (*77*). In the future, TETRIS can provide a standardized, quantifiable and (with the help of artificial intelligence) autonomous solution to accelerate plant phenotyping. TETRIS has the potential to overcome the urgent “bottleneck” in high-throughput screening in producing high yielding plant (*78*).

## MATERIALS AND METHODS

### Plant growth

A hydrophobic wax border was printed (Xerox ColorQube 8580) onto chromatography paper (Whatman, grade 1 chromatography paper, 0.18 mm in thickness) and heat-transferred (Vevor HP230B) to define a circular area (radius: 17.5 mm; area: 962 mm^2^). Seeds were prepared and germinated: *B. oleracea acephala* (“dwarf green curled kale,” Mr. Fothergill’s) seeds were stirred in 3% hydrogen peroxide (Millipore) for 5 min, rinsed in deionized water, and germinated on damp tissue paper for 2 days before transferring onto the wax-printed chromatography paper discs; *S. lycopersicum* L. (tomato Heinz 1350, Chiltern Seeds) seeds were stirred in 30% commercial bleach, 0.02% Tween-20 (Sigma-Aldrich) for 15 min, rinsed in deionized water, and germinated on damp tissue paper for 4 days before transfer; *O. sativa* (rice Black Madras, Kings Seeds) seeds were stirred in 3% hydrogen peroxide for 15 min, rinsed in deionized water, and germinated on damp tissue paper for 1 week before transfer. The paper discs with seedlings were placed onto platforms inside a propagator box with transparent lid, and the paper was constantly supplied with deionized water from a reservoir through paper strips. For experiments with seedlings grown in multinutrient solution, the deionized water in the growth box was substituted with diluted Miracle-Gro (Evergreen Garden Care UK Ltd., purchased from the local garden center). For experiments using agar as the plant growth medium, sensors were fixed into the petri dish base and 0.6% agar (Sigma-Aldrich) in deionized water filled to surface. Pregerminated seeds were placed on the agar surface and the petri dish was placed into the growth box for 1 week.

### General experimental setup

During measurements, the sensor was adhered to the base of a petri dish (55 mm in diameter), placed into a measurement chamber consisting of a silicone base with a reservoir of water and connected to a potentiostat with crocodile clips. A transparent colorless acrylic lid was placed over the experimental setup, and the impedance of the sensor with the sample placed on top was measured. All electrochemical experiments were performed using a PalmSens4 potentiostat and MUX8-R2 multiplexers.

### Humidity and temperature of measurement chamber

The relative humidity and temperature were measured inside and outside of the measurement chambers using the Adafruit BME280 (Bosch) I2C or SPI Temperature Humidity Pressure Sensor connected to an Arduino Due and logged using PuTTY.

### Impedance sensor fabrication and experimental setup

Carbon electrodes {Sun Chemical C2130925D1 conductive carbon ink [80 weight % (wt %)] and Gwent Group S60118D3 diluent (20 wt %)} were screen-printed onto a polyester transparency sheet (Office Depot). The sensor design consisted of two identical electrodes (30 mm by 6.25 mm) separated by 5 mm (Fig. 2). Impedance measurements [amplitude 0.25 V_half wave_ (root mean square), frequency 2 kHz, 0 V dc] were performed.

### Impedance sensor characterization

#### Calibration for each salt

The impedance was measured between 0.125 and 1.00 mM (1 ml, *n* = 8) for the following compounds on paper discs: BaCl_2_, CaCl_2_, Ca(NO_3_)_2_, CdCl_2_, Cd(NO_3_)_2_, CdSO_4_, Cu(NO_3_)_2_, CuSO_4_, GdCl_3_, K_2_SO_4_, LaCl_3_, Mg(NO_3_)_2_, MgCl_2_, Na_2_CO_3_, Na_2_SO_4_, NaOH, Ni(NO_3_)_2_, and NiCl_2_ (salts were purchased from Sigma-Aldrich). An extended range (0.01 μM to 0.1 M, *n* ≥ 15) was measured for the following salts: AgNO_3_, CuCl_2_, KCl, KH_2_PO_4_, KNO_3_, NaCl, NaH_2_PO_4_, NaNO_3_, NH_4_Cl, NH_4_H_2_PO_4_, and NH_4_NO_3_. Impedance was measured for at least 2 hours, and the average of the impedance response was recorded. Log_10_(*Z*) was plotted against log_10_(*c*) and the linear fit was found for each salt.

#### Response time

The impedance of a paper disc with 270 μl of deionized water was measured. After 60 s, 30 μl of KCl (0.1 M) was added.

#### Effect of volume

The impedance of a paper disc with 125 to 1000 μl of KCl (0.1 M) was measured for at least 3000 s.

#### Electrochemical impedance spectroscopy

Experiments were run at 0 V dc and 0.25 V ac. For EIS with kale seedlings, the disc with seedlings was placed on the sensor with no added deionized water. For EIS in 0.1 M KCl, the sensor was placed in a solution of 10-ml volume.

### pH sensor fabrication, experimental setup, and characterization

Carbon [Sun Chemical C2130925D1 conductive carbon ink (80 wt %) and Gwent Group S60118D3 diluent (20 wt %)] and silver/silver chloride [Sun Chemical C2130809D5 (95 wt %) and Gwent Group diluent S60530D5 (5 wt %)] electrodes were screen-printed onto a polyester transparency sheet (Office Depot). Polyaniline was electropolymerized (1 V dc, 100 min) from a solution of aniline (0.1 M) and oxalic acid (0.3 M) onto the carbon electrode to form the working electrode (WE) (sensing area: 2 mm by 40 mm). The silver/silver chloride electrode was the reference electrode (RE) (sensing area: 2 mm by 18 mm). Open circuit potentiometric measurements were performed. Characterization experiments were performed in 1 M KCl in bulk solution (not on paper). The pH was altered by the addition of H_2_SO_4_ and NaOH. pH was measured using a Hanna Instruments HI 5521 pH meter.

### H_2_O_2_ sensor fabrication, experimental setup, and characterization

Prussian blue–mediated carbon [Sun Chemical C2070424P2 (80 wt %) and Gwent Group S60118D3 diluent (20 wt %)], carbon [Sun Chemical C2130925D1 (80 wt %) and Gwent Group S60118D3 diluent (20 wt %)], and silver/silver chloride [Sun Chemical C2130809D5 (95 wt %) and Gwent Group diluent S60530D5 (5 wt %)] electrodes were screen-printed onto a polyester transparency sheet (Office Depot). The Prussian blue–mediated carbon WE (sensing area: 2 mm by 15 mm) was placed between the carbon counter electrode and Ag/AgCl RE (sensing area: 2 mm by 18 mm each) with a gap of 1 mm. Amperometric measurements were performed at 0 V. Characterization experiments were performed in KCl (1 M, 1 ml) on paper.

### H_2_O_2_ plant experiments

For the external addition of H_2_O_2_ experiment, KCl (2.2 M, 136.4 μl) was added to the paper. Current was measured over time at 0 V relative to the printed RE and H_2_O_2_ (500 μM, 30 μl, in 1 M KCl) was added to the paper after at least 1 hour.

For the mechanical damage experiments, mechanical damage to plants was performed by gently slicing the roots with a sterilized scalpel. Mechanical damage to paper control was performed by gently slicing the paper with a sterilized scalpel.

### Combined pH and impedance plant experiments

A paper disc with 30 seedlings was removed from growth chamber 9 days after germination and placed onto the sensor module in the measurement chamber, where the pH and impedance sensors were placed at least 10 mm apart. One hundred microliters of deionized water was added to the paper. Impedance and pH were measured over time, and the pH of the paper was altered by the addition of NaOH (pH 9, 300 μl).

### Salt uptake experiments

A disc with seedlings was removed from the growth chamber after a set number of days and placed onto the sensor in the measurement chamber, and 100 μl of deionized water was added to the paper. After measuring impedance for at least 3 hours, 30 μl of salt solution was added to the paper through a port in the lid. To calculate the rate of uptake, impedance was converted to salt concentration by the obtained calibration curves. An initial baseline concentration was found by taking the average concentration of the first 2 hours after measurement began. The time where the concentration was highest after addition of salt was found, and a logarithmic curve (of the form *c* = *Bt*^*k*^) was fitted from this time. Where the curve would meet the initial baseline (with a 5% tolerance), the constant *k* of the curve was found up to that time point. Where the line did not reach the initial baseline, a curve was plotted until the end of the experiment, and the constant *k* was found. Where the concentration did not decrease after the addition of salt (and therefore, there was no maximum concentration), the uptake was set to zero. The constant *k* found for each experiment was then divided by the average *k* found for the control (no plants) to find the normalized rate of uptake.

### Ion channel blocker experiments

The paper discs with seedlings were removed from the water reservoir after 7 days. Thirty microliters of blocker and 100 μl of deionized water were added to the paper. After 4 hours, the discs with seedlings were washed in deionized water, and the seedlings were transferred to fresh prewetted discs of paper in the measurement chamber. Two hundred microliters of deionized water was added to the paper. After measuring impedance for at least 24 hours, 30 μl of salt solution was added to the paper through a port in the lid.

### Conductivity of stem samples

Following on from salt uptake experiments, stem samples were tested using a simultaneous multiwell impedance measurement system developed for electrolyte leakage assays (*45*). The system consisted of an array of measurement wells, each with a two-electrode assembly interfaced with external electronics that facilitated the measurement of solution conductivity in each well. Three 1-cm cuts of stem (measured from the base of the stem upward) from the same salt treatment group were placed in each individual well in 2 ml of distilled water. The conductivity of each solution was sampled every 2 min over a 40-hour period using an excitation frequency of 10 kHz.

## Supplementary Materials

### This PDF file includes:

Supplementary Text Figs. S1 to S10 Tables S1 to S3 Legends for movies S1 and S2 References
