# Supplementary material for: Time-resolved chemical monitoring of whole plant roots with printed electrochemical sensors and machine learning
Source: Sci Adv. 2024 Jan 31;10(5):eadj6315. doi: 10.1126/sciadv.adj6315 (PMC10830104; doi:10.1126/sciadv.adj6315)
Supplement: Supplementary file 1 — Supplementary Text Figs. S1 to S10 Tables S1 to S3 Legends for movies S1 and S2 References [file sciadv.adj6315_sm.pdf]

Science Advances
AAAS

Supplementary Materials for

Time-resolved chemical monitoring of whole plant roots with printed electrochemical sensors and machine learning

Philip Coatsworth *et al.*

Corresponding author: Firat Güder, guder@imperial.ac.uk; Philip Coatsworth, p.coatsworth18@imperial.ac.uk

Sci. Adv. **10**, eadj6315 (2024)
DOI: 10.1126/sciadv.adj6315

The PDF file includes:

Supplementary Text
Figs. S1 to S10
Tables S1 to S3
Legends for movies S1 and S2
References

Other Supplementary Material for this manuscript includes the following:

Movies S1 and S2

Supplementary text

Characterization of sensors

We designed three sensors for use in TETRIS: i) an impedance-based non-specific salt concentration sensor, ii) a potentiometric pH sensor and iii) an amperometric H_2O_2 sensor (Figure 1B-E, **Figure S3**). Our low-cost (~$0.02 per sensor) salt concentration sensor consisted of two screen-printed carbon electrodes on polyester transparency sheet. By measuring the impedance of the solution between the carbon electrodes with an excitation signal of 2 kHz, 0.25 $V_{\text{half wave}}$ (RMS), we were able to monitor the overall, non-specific salt concentration in the paper substrate. Electrical impedance measurements were chosen due to non-ion-specificity (unlike a sensor with an ion-sensitive membrane, for example).(*79*, *80*) The chosen frequency was selected as it produced good differentiation between salt concentrations in the ranges appropriate for the experiments described in this article. Two electrical impedance spectroscopy experiments were performed (Figure 1B, Figure 2B), where impedance is measured over a frequency sweep, to demonstrate the compatibility of TETRIS with EIS. While beyond the scope of our work here, EIS has already been used to study the effects of abiotic stress (salt, alkali and cadmium), cell growth and tissue damage in plant seedlings and tissues, and the combination of EIS with the other sensors in TETRIS could expand this research in the future.(*81*, *82*)

Calibration experiments for the impedance-based sensor were carried out for salts consisting of a range of cations (Ag^+, Ba^{2+}, Ca^{2+}, Cd^{2+}, Cu^{2+}, Gd^{3+}, K^+, La^{3+}, Mg^{2+}, Na^+, Ni^{2+}, NH_4^+) and anions (Cl^-, CO_3^{2-}, PO_4^{3-}, NO_3^-, OH^-, SO_4^{2-}) on paper discs. A relationship was found between the logarithm of total electrical impedance (Z) and the logarithm of salt concentration (c), where impedance decreased with increasing salt concentration. Calibration curves were found for all the salts by taking measurements from 0.125–1.00 mM (**Table S1**). Extended

studies for some salts (Table S1) demonstrate that this log(*c*)-log(*Z*) relationship extends down to 0.1 mM and up to 0.1 M, as shown for KCl in **Figure 2B**. The response time of the sensor after the addition of salt to paper was fast (faster than the frequency of measurement the potentiostat allowed), followed by a lengthier stabilization time before a steady impedance value was reached, likely due to ion diffusion through the paper (Figure S3).(*83*) Addition of deionized water did not have any observable effects on the overall impedance of paper that was already wet. Differences in total volume of solution in the paper were found to have only a small effect on the measured impedance, with considerably higher impedance only observed for low volumes (<200 µL), where substantial evaporation occurred (Figure S3) within hours (<5 hours until visibly dry) for these smaller volumes. This emphasized the importance for a controlled measurement chamber with a constant relative humidity to prevent evaporation and therefore erroneous measurements. The silicone base, acrylic lid and water reservoir did slow down evaporation in the paper, although some evaporation continued to occur in the experiments, leading to a slight up-trending drift in impedance over time. We were able to achieve good reproducibility between sensors for KCl concentrations in the range of 0–0.01 M (standard deviation between values for each concentration ≤ 0.083 for log(*Z* / Ohm), n = 5 each at five concentrations) as shown in Figure S3. The sensors also showed good reversibility when increasing and decreasing KNO_3 concentration (Figure S3).

The pH sensor produced for TETRIS consisted of two screen-printed electrodes. pH was measured with open-circuit potentiometry, which measures the open-circuit voltage between two electrodes, once sensitive to protons in solution. The working electrode (WE) consisted of printed carbon with a layer of polyaniline (PANI) electropolymerized onto the electrode surface. PANI facilitated pH measurement through protonation and deprotonation of nitrogen atoms on

the polymer chain with decreasing and increasing pH, respectively, leading to a change in surface charge and, therefore, electrical potential. The reference electrode (RE) was formed of silver/silver-chloride ink (60:40). We found that our pH sensor produced a linear response of -67.5 ± 1.7 mV per pH (r^2 = 0.973) between pH 2.7 and 10.5 in 1 M KCl (**Figure 2C**), comparable to other printed pH sensors in the literature.(*20*, *21*) This slightly super-Nernstian response has previously been reported in PANI-based pH sensors, possibly due to surface hydration effects at time of formation of the PANI layer.(*20*, *84*, *85*) Our sensors displayed long-term stability suitable for our use-case (where the sensors were used for up to ~100 hours), with an average drift of only 0.68 mV $hour^{-1}$, or -0.010 pH $hour^{-1}$ (Figure S3). While calibration experiments were performed in bulk solution to enable the use of a standard pH electrode, we observed the same pH behavior for our sensors using paper disc substrates.

The H_2O_2 sensor in TETRIS consisted of three screen-printed electrodes: a Prussian blue-mediated carbon WE, a Ag/AgCl RE and a carbon counter electrode (CE), measured amperometrically at 0 V *vs* the printed pseudo-RE. The calibration experiment was performed in 1 ml of 1 M KCl on the paper discs. We found a sensitivity of -0.24 ± 0.02 μA μM^{-1} between 2 and 100 μM H_2O_2 for our sensor with a WE geometric area of 30 mm^2 (**Figure 2D**). The measurements produced a linear relation between the current reading and concentration which is in agreement with the previous reports in the literature for this system.(*73*) We observed larger variations in the signals measured at higher concentrations (standard deviation = 6.3 μA at 100 μM H_2O_2, 0.32 μA at 1 μM H_2O_2), possibly due to variations in the geometry of the electrodes between each sensor and variation in the diffusion of H_2O_2 through the paper. Extracellular H_2O_2 concentration due to salt stress, metals or other chemical stimuli can change in the order of 0.1 μM to tens of μM, a range covered partly but not fully by our sensor, and the

concentration in the growth media (for example, soil or agar) is likely to be even less.(*86*) To further increase the sensitivity, we could potentially roughen the electrode surface to increase surface area, deposit nanoparticles of Prussian Blue or platinum, or fully optimize the appropriate voltage.(*22*, *23*) Constraints remain in the general setup, however, where diffusion of H_2O_2 is lowered by the paper substrate and could provide a limit of detection higher than the sensor would otherwise be capable of.

Prediction of salt uptake with machine learning

XGBoost was used to predict the rate of uptake of salts on the following input variables: 1) cation, 2) anion, 3) cation classification (heavy metal, primary macronutrient, secondary macronutrient, micronutrient, sodium), 4) cation wider chemical classification (transition metal, s-block, lanthanum, none), 5) cation chemical group, 6) cation chemical period, 7) cation charge, 8) number of anions, 9) anion charge, 10) anion classification (primary macronutrient, secondary macronutrient, micronutrient, neutral), 11) cation relative mass, 12) anion relative mass, 13) total salt mass, 14) Salt solubility at 25 °C. The categorical features (1-10) were encoded as a one-hot numeric array. For all models, exhaustive searches were performed to identify optimal hyperparameters used, by a 5-fold cross-validated grid-search over a parameter grid. The hyperparameters used for the classification models were as follows: 6 classes (objective: "multi:softprob", booster: "gbtree", eval_metric: "merror", eta: 0.1, max_depth: 3, subsample: 0.7, colsample_bytree: 0.6); 5 classes (objective: "multi:softprob", booster: "gbtree", eval_metric: "merror", eta: 0.1, max_depth: 7, subsample: 0.9, colsample_bytree: 0.6); 4 classes (objective: "multi:softprob", booster: "gbtree", eval_metric: "merror", eta: 0.1, max_depth: 3, subsample: 0.6, colsample_bytree: 0.4); 3 classes

(objective: "multi:softprob", booster: "gbtree", eval_metric: "merror", eta: 0.15, max_depth: 3, subsample: 0.7, colsample_bytree: 0.1); 2 classes (objective: "binary:logistic", booster: "gbtree", eval_metric: "logloss", eta: 0.15, max_depth: 5, subsample: 0.6, colsample_bytree: 0.6).

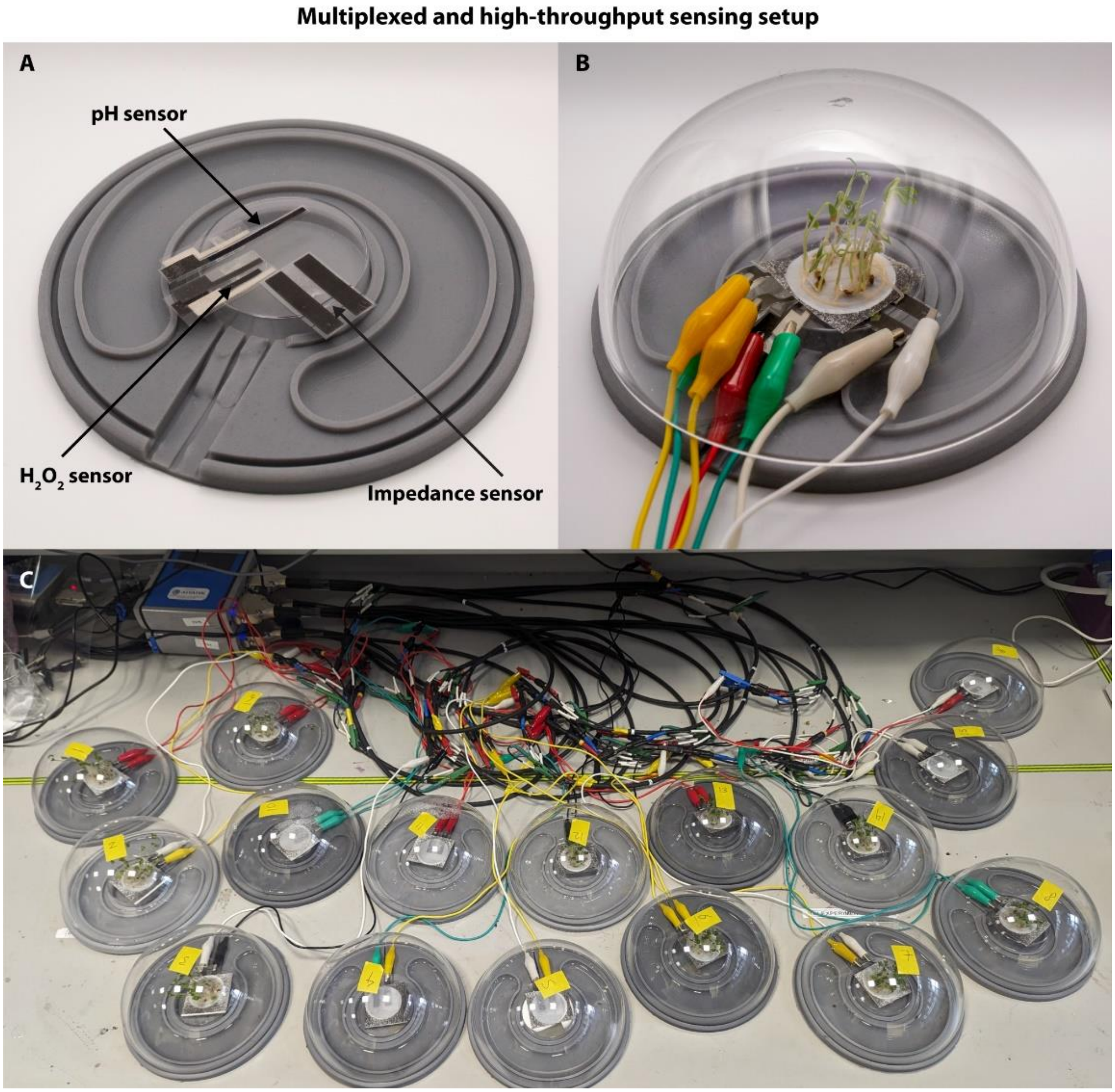

Fig. S1.

Photographs showing the multiplexed and high-throughput setup of TETRIS. (**A**) The three sensors (impedance salt concentration sensor, PANI-based pH sensor and Prussian Blue-based H_2O_2 sensor) placed laterally on the sensing module, fixed into the silicone base. (**B**) The same setup as a), with seedlings on paper and acrylic lid placed on top, sensors connected to a potentiostat. (**C**) 16 multiplexed impedance experiments running simultaneously.

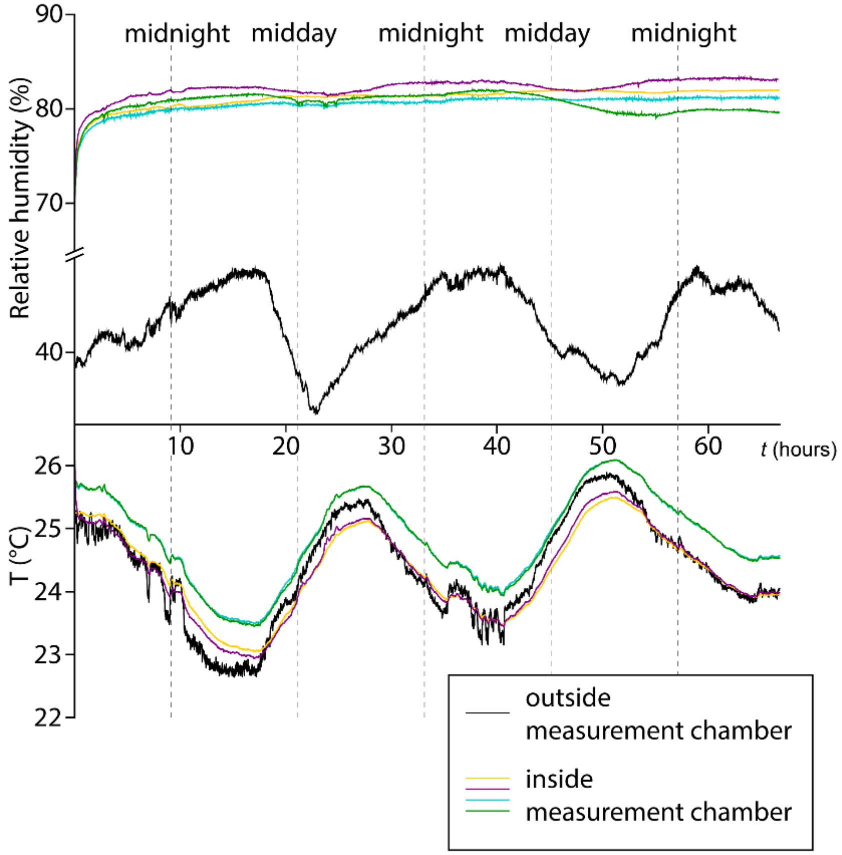

Fig. S2.

Humidity and temperature recordings for TETRIS. The relative humidity (top) and temperature (bottom) measured inside (yellow, purple, teal and green lines) and outside (black line) the measurement chamber, demonstrating the stability of the internal relative humidity despite changing temperature and external relative humidity. Each internal measurement was performed in an individual measurement chamber. A standard salt uptake experiment with kale seedlings was performed during these measurements, where 30 µL 0.1 M KCl was added through the measurement port at least 2 hours after measurements began; no obvious change in humidity was detected during this treatment step.

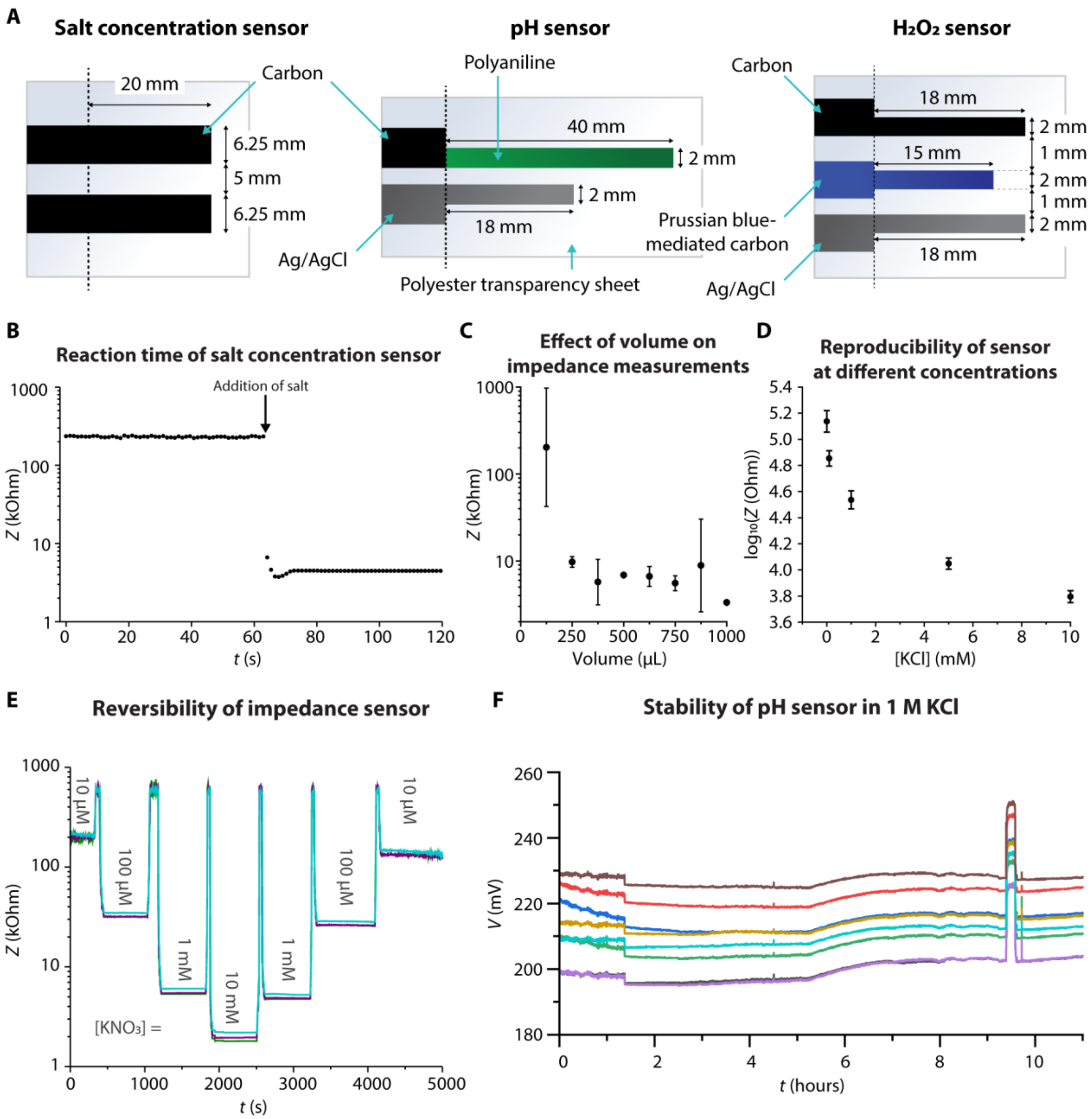

Fig. S3.

Additional characterization of TETRIS. (**A**) Dimensions and materials of our three sensors. (**B**) Sensor response to sudden change in salt concentration. Arrow indicates time of addition of KCl (0.1 M, 30 µL) to paper disc containing 270 µL deionized water. (**C**) Effect of volume of KCl (0.1 M) on impedance measurements. Error bars indicate 1 standard deviation (n = 2). (**D**) Reproducibility of salt sensors at different concentrations. The logarithm of impedance of printed sensor with 500 µL solution in paper disc, at KCl concentrations of 0, 0.1, 1, 5 and 10 mM. Error bars show standard deviation (n = 5). (**E**) Reversibility of impedance sensor to KNO_3 concentrations from 10 µM to 10 mM. (**F**) Stability of our pH sensor in 1 M KCl.

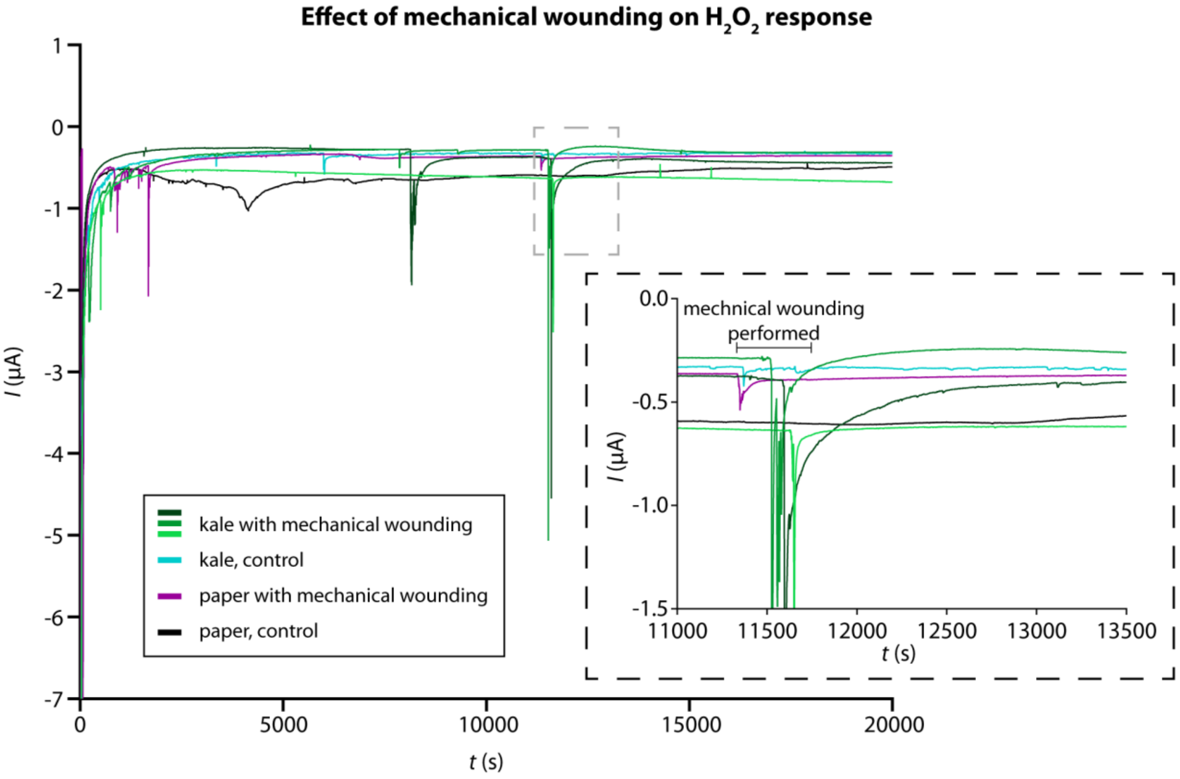

Fig. S4.

Effect of mechanical wounding on H_2O_2 response of plants. Amperometric response of our Prussian Blue-mediated H_2O_2 sensor upon mechanical wounding to the roots of kale seedlings (9-days-old, 30 seedlings, green signals). Controls included kale with no wounding (cyan), paper-only with (purple) and without (black) mechanical stimulation.

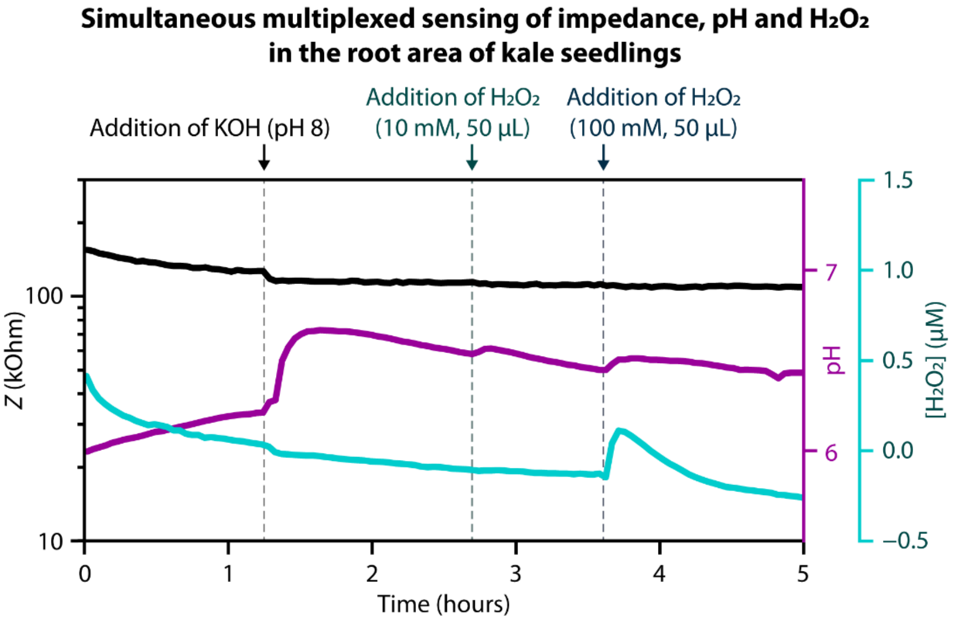

Fig. S5.

Simultaneous multiplexed sensing of impedance, pH and H_2O_2 in the root area of kale seedlings. Real-time monitoring of impedance (black), pH (purple) and [H_2O_2] (cyan) of the root environment of kale seedlings (30 plants, 9-days-old) MiracleGro nutrient solution (6.4 mM). Dilute KOH (pH 8, 100 µL) and H_2O_2 (10 and 100 mM, 50 µL) were added 1.25, 2.7 and 3.6 hours, respectively. The impedance signal showed a small decrease upon addition of KOH (pH 8) due to addition of ions to the solution, but did not display any changes upon addition of H_2O_2, a molecule with no constituent ions. The pH-sensitive polyaniline layer on the pH sensor facilitated greater change in signal upon addition of KOH, compared to only small change upon addition of H_2O_2, due to the pH selectivity of polyaniline. Likewise, the H_2O_2-sensitive Prussian Blue mediated sensor showed greater response with addition of H_2O_2 (100 mM, 50 µL) than with change in pH. The first addition of 10 mM H_2O_2 did not give a notable response in A, likely due to the large amount of solution present due to the KOH addition, physical blocking by the roots and enzymes produced by the roots removing H_2O_2.

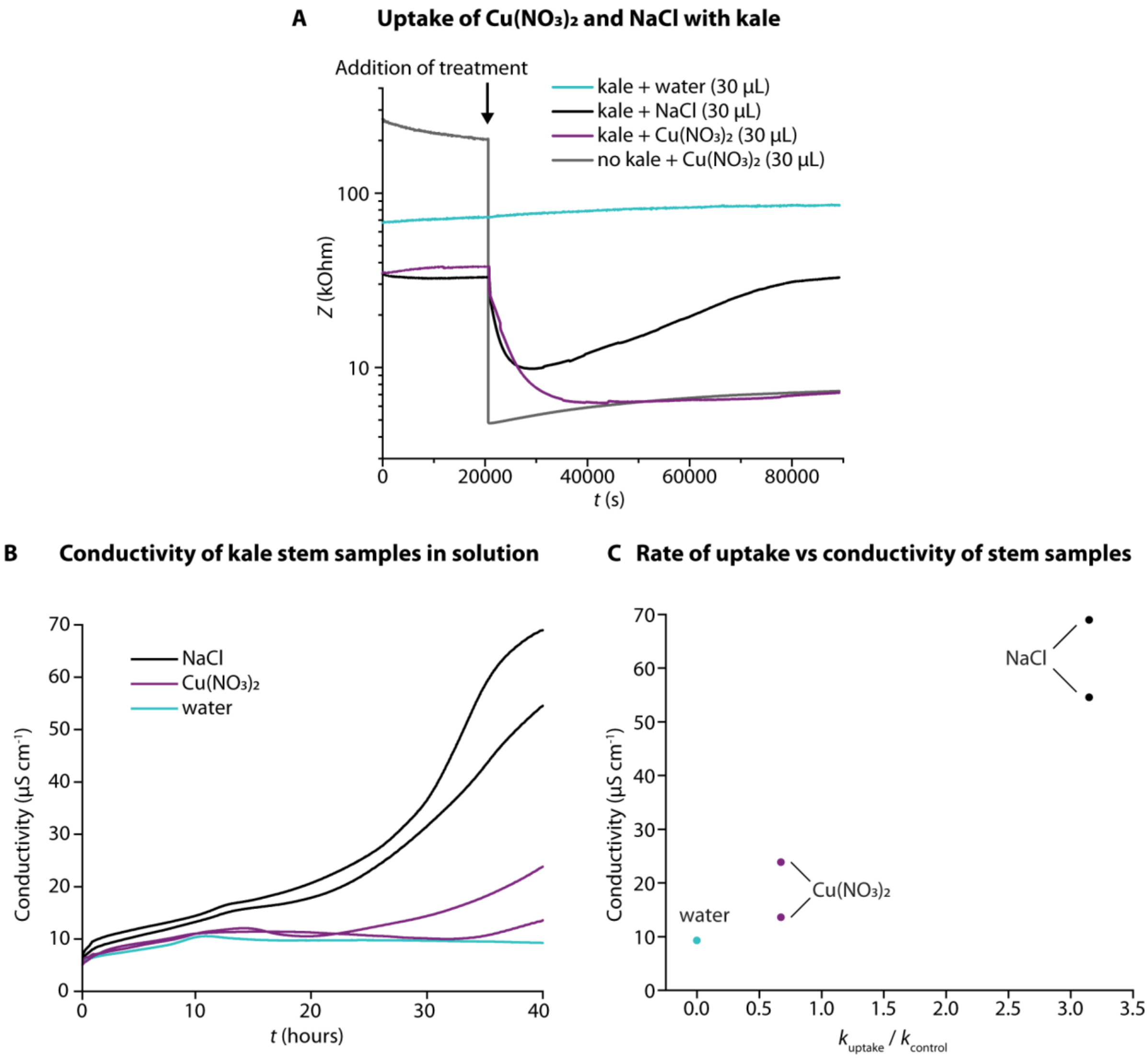

Fig. S6.

Electrolyte assay test. (**A**) Uptake of $Cu(NO_3)_2$ and NaCl (addition of 0.1 M, 30 μL) or deionized water (30 μL) by 30 kale seedlings, with no plant control. (**B**) The conductivity of samples of stem taken from plants treated with NaCl, $Cu(NO_3)_2$ or deionized water. (**C**) The rate of uptake of ions with the corresponding conductivity of stem samples.

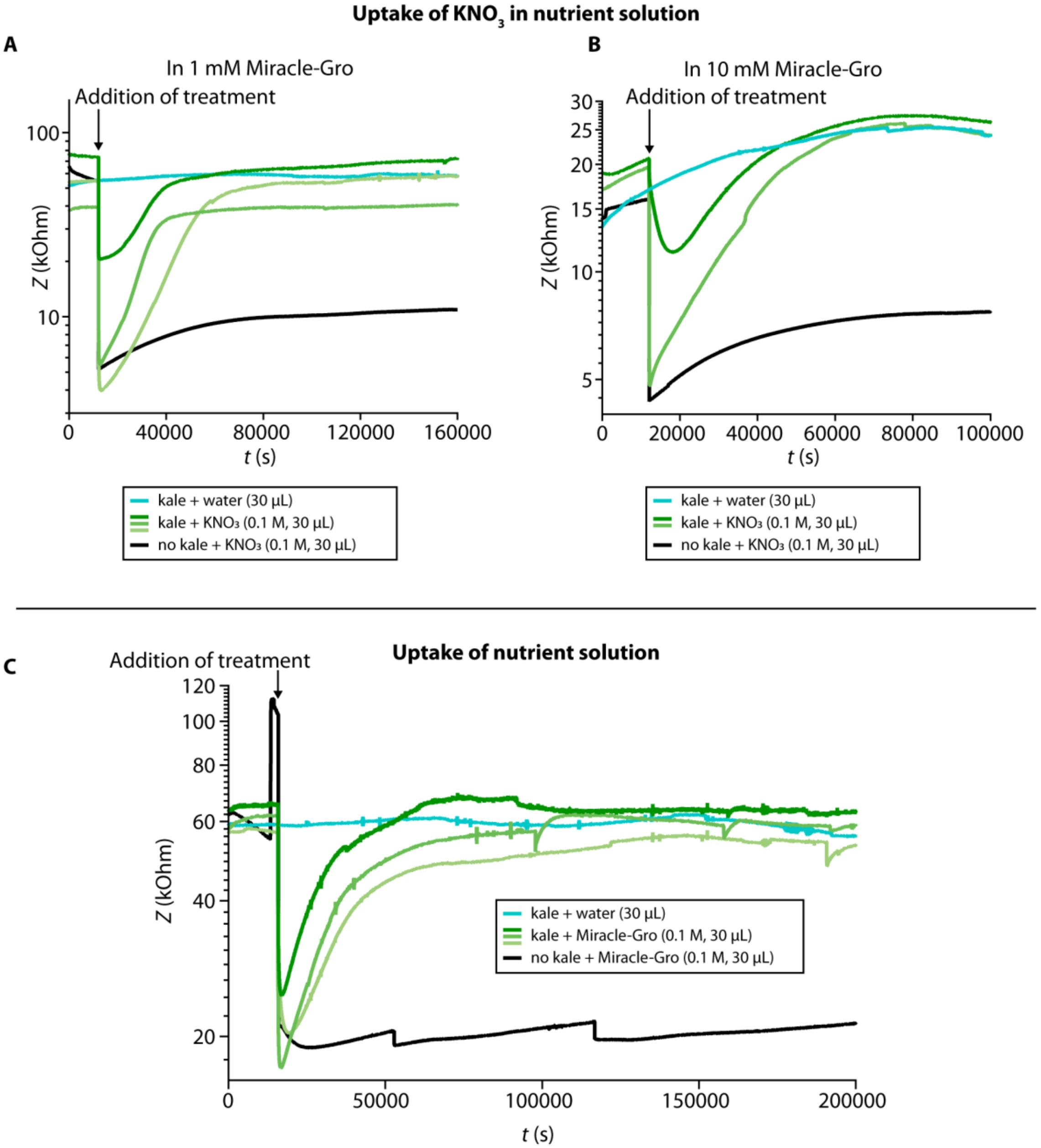

Fig. S7.

Compatibility of TETRIS with multi-nutrient solution. Uptake of KNO_3 (addition of 0.1 M, 30 µL) can be detected with 30 kale seedlings (green signals) compared to addition of water (30 µL) to 30 kale seedlings (cyan) and to addition of KNO_3 (0.1 M, 30 µL) to paper with no kale seedlings (black), grown in solutions of Miracle-Gro (commercial multi-nutrient plant food) at total ion concentrations of both (**A**) 1 mM and (**B**) 10 mM. (**C**) Uptake of added Miracle-Gro (total ion concentration 0.1 M, 30 µL) by 30 kale seedlings (green signals) grown in deionized water, compared to addition of Miracle-Gro (total ion concentration 0.1 M, 30 µL) to paper with no kale seedlings (black) and addition of water (30 µL) to 30 kale seedlings (cyan).

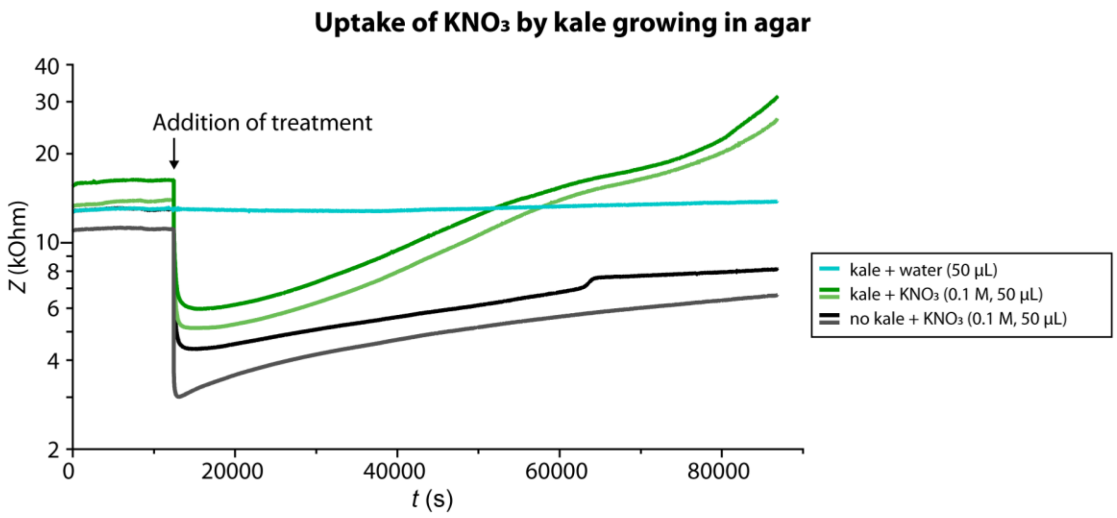

Fig. S8.

Compatibility of TETRIS with agar. Uptake of KNO_3 (addition of 0.1 M, 30 µL) can be detected with 30 kale seedlings grown in agar medium (green signals) compared to addition of water (30 µL) to 30 kale seedlings (cyan), to addition of KNO_3 (0.1 M, 30 µL) to agar with no kale seedlings (black), and to addition of water (30 µL) to agar with no kale seedlings. Substantial evaporation began to occur after 20 hours.

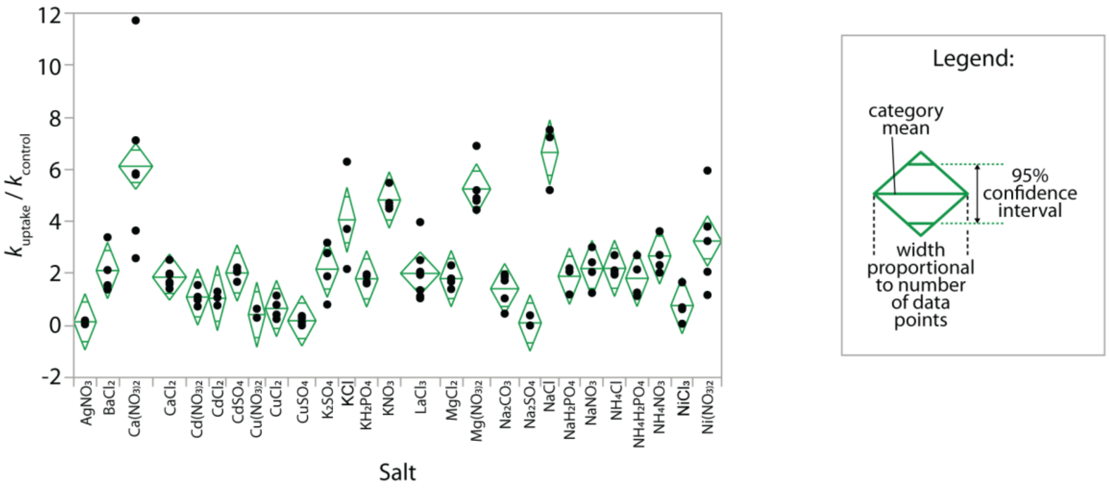

Fig. S9.

Statistical analysis between uptake of each salt. One-way ANOVA statistical analysis showed significant differences in uptake between some of the salts, where confidence diamonds show mean uptake, 95% confidence interval and number of data points.

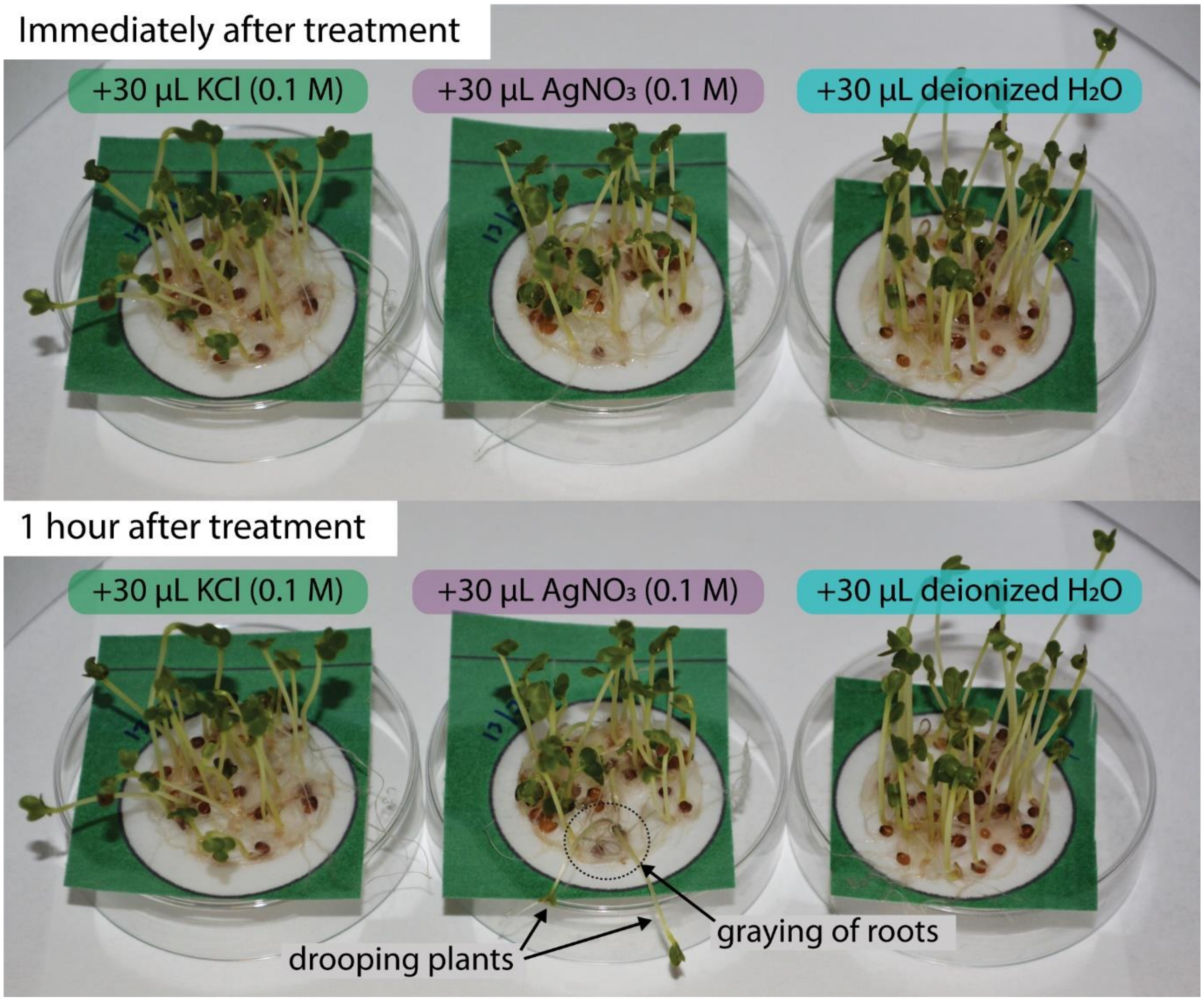

Fig. S10.

Visual effect of $AgNO_3$ treatment. Treating kale seedlings with $AgNO_3$ solution resulted in metallic silver precipitating onto the roots, leaving a gray coloration. Some drooping of the plants was also observed for those plants where the gray coloration was the most obvious. No color change was observed with treatment of KCl or water.

Table S1.

Calibration of impedance sensor for different salts. The constants *A* and *n* and adjusted R-squared value of the line of best fit from plotting log(*Z*) against log(*c*), where the relationship of electrical impedance (*Z*) and salt concentration (*c*) had the form $Z=Ac^n$.

**Salt**	***A***	***n***	**Adj. R-squared**	**Linear range found**	**Number of measurements in linear range**
$AgNO_3$	304.1865	-0.65131	0.80214	0.1 mM–0.1 M	15
$BaCl_2$	142.8828	-0.64357	0.80206	0.125–1 mM	8
$Ca(NO_3)_2$	258.6128	-0.59552	0.767	0.125–1 mM	8
$CaCl_2$	2.46082	-1.32354	0.87006	0.125–1 mM	8
$Cd(NO_3)_2$	163.7948	-0.63987	0.90484	0.125–1 mM	8
$CdCl_2$	334.349	-0.56173	0.85033	0.125–1 mM	8
$CdSO_4$	6704.401	-0.37509	0.90133	0.125–1 mM	8
$Cu(NO_3)_2$	118.3123	-0.67205	0.98613	0.125–1 mM	8
$CuCl_2$	286.7742	-0.5752	0.971	0.1 mM–0.1 M	15
$CuSO_4$	383.0893	-0.53075	0.98461	0.125–1 mM	8
$GdCl_3$	433.6107	-0.48196	0.76721	0.125–1 mM	8
K_2SO_4	5.92611	-1.0773	0.93764	0.125–1 mM	8
KCl	291.1052	-0.62373	0.93281	0.1 mM–0.1 M	15
KH_2PO_4	363.5465	-0.62948	0.93313	0.1 mM–0.1 M	12
KNO_3	329.9286	-0.58908	0.80775	0.1 mM–0.1 M	15
$LaCl_3$	148.6004	-0.6505	0.8656	0.125–1 mM	8
$Mg(NO_3)_2$	580.50471	-0.4769	0.85103	0.125–1 mM	8
$MgCl_2$	85.29233	-0.70649	0.96773	0.125–1 mM	8
Na_2CO_3	429.32078	-0.51237	0.82423	0.125–1 mM	8
Na_2SO_4	204.9085	-0.63517	0.92592	0.125–1 mM	8
NaCl	214.9661	-0.69028	0.90808	0.1 mM–0.1 M	23
NaH_2PO_4	420.5329	-0.60364	0.93292	0.1 mM–0.1 M	12
$NaNO_3$	492.7765	-0.59425	0.92538	0.1 mM–0.1 M	12
NaOH	0.11724	-1.59736	0.84581	0.125–1 mM	8
NH_4Cl	339.8365	-0.57598	0.886	0.1 mM–0.1 M	12
$NH_4H_2PO_4$	448.3632	-0.61392	0.96546	0.1 mM–0.1 M	12
NH_4NO_3	185.2593	-0.64442	0.78426	0.1 mM–0.1 M	12
$Ni(NO_3)_2$	187.17723	-0.60573	0.86894	0.125–1 mM	8
$NiCl_2$	187.5858	-0.61078	0.83476	0.125–1 mM	8

Table S2.

Composition of Miracle-Gro multi-nutrient solution.

**Component**	**Percentage of composition**
Nitrogen (N) total	7.0%
Urea nitrogen (N)	3.5%
Nitric nitrogen (N)	1.7%
Ammoniacal nitrogen (N)	1.8%
Phosphorus pentoxide (P_2O_5) soluble in water	3.0% (1.3% P)
Potassium oxide (K_2O) soluble in water	5.0% (4.3% K)
Copper (Cu), water soluble, chelated by EDTA	0.002%
Iron (Fe), water soluble, chelated by DTPA	0.03%
Manganese (Mn), water soluble, chelated by EDTA	0.01%
Molybdenum (Mo) soluble in water	0.001%
Zinc (Zn), water soluble, chelated by EDTA	0.002%

Table S3.

Statistical analysis between uptake of each salt. Tukey-Kramer HSD post-hoc test showed significant pairwise differences between some of the salts. Salts not connected by same letter are significantly different.

**Salt**							**Mean uptake**
NaCl	A						6.650131
$Ca(NO_3)_2$	A						6.117539
$Mg(NO_3)_2$	A	B					5.246398
KNO_3	A	B	C				4.819403
KCl	A	B	C	D			4.057744
$Ni(NO_3)_2$		B	C	D	E		3.246344
NH_4NO_3		B	C	D	E	F	2.671568
NH_4Cl			C	D	E	F	2.195178
$NaNO_3$			C	D	E	F	2.186407
K_2SO_4			C	D	E	F	2.164408
$BaCl_2$			C	D	E	F	2.112961
$CdSO_4$			C	D	E	F	2.016057
$LaCl_3$				D	E	F	2.003104
NaH_2PO_4			C	D	E	F	1.890649
$CaCl_2$				D	E	F	1.85847
$NH_4H_2PO_4$				D	E	F	1.812488
$MgCl_2$				D	E	F	1.800555
KH_2PO_4				D	E	F	1.791352
Na_2CO_3				D	E	F	1.412822
$Cd(NO_3)_2$				D	E	F	1.094569
$CdCl_2$				D	E	F	1.0494
$NiCl_2$					E	F	0.764495
$CuCl_2$					E	F	0.653345
$Cu(NO_3)_2$					E	F	0.415253
$CuSO_4$						F	0.18067
$AgNO_3$						F	0.139697
Na_2SO_4						F	0.097677

Movie S1.

Our multiplexed sensing system, TETRIS, consisting of sensors in stacked or lateral positioning, seedlings grown on chromatography paper and an enclosed measurement chamber. The sensors can be used in parallel to monitor real-time effects of chemical treatments or abiotic stressors in the root area of the plant.

Movie S2.

Setup and experimental procedure for continuous measurement of uptake of salt using our sensing system TETRIS. Single-sensor and multiplexed use is shown.

REFERENCES AND NOTES

1. R. Pieruschka, U. Schurr, Plant phenotyping: Past, present, and future. *Plant Phenomics* **2019**, 7507131 (**2019**).

2. H. H. Felle, F. Waller, A. Molitor, K.-H. Kogel, The mycorrhiza fungus *Piriformospora indica* induces fast root-surface pH signaling and primes systemic alkalinization of the leaf apoplast upon powdery mildew infection. *Mol. Plant Microbe Interact.* **22**, 1179–1185 (2009).

3. Z. Xu, G. Zhou, H. Shimizu, Plant responses to drought and rewatering. *Plant Signal. Behav.* **5**, 649–654 (2010).

4. X. R. R. Sirault, R. A. James, R. T. Furbank, A new screening method for osmotic component of salinity tolerance in cereals using infrared thermography. *Funct. Plant Biol.* **36**, 970–977 (2009).

5. S. Fujimaki, T. Maruyama, N. Suzui, N. Kawachi, E. Miwa, K. Higuchi, Base to tip and long-distance transport of sodium in the root of common reed [*Phragmites australis* (Cav.) Trin. ex Steud.] at steady state under constant high-salt conditions. *Plant Cell Physiol.* **56**, 943–950 (2015).

6. S. K. Gjetting, C. K. Ytting, A. Schulz, A. T. Fuglsang, Live imaging of intra- and extracellular pH in plants using pHusion, a novel genetically encoded biosensor. *J. Exp. Bot.* **63**, 3207–3218 (2012).

7. M. Toyota, D. Spencer, S. Sawai-Toyota, W. Jiaqi, T. Zhang, A. J. Koo, G. A. Howe, S. Gilroy, Glutamate triggers long-distance, calcium-based plant defense signaling. *Science* **361**, 1112–1115 (2018).

8. M. Sadoine, Y. Ishikawa, T. J. Kleist, M. M. Wudick, M. Nakamura, G. Grossmann, W. B. Frommer, C. H. Ho, Designs, applications, and limitations of genetically encoded fluorescent sensors to explore plant biology. *Plant Physiol.* **187**, 485–503 (2021).

9. P. Coatsworth, L. Gonzalez-Macia, A. S. P. Collins, T. Bozkurt, F. Güder, Continuous monitoring of chemical signals in plants under stress. *Nat. Rev. Chem.* **7**, 7–25 (2023).

10. M. Janni, C. Cocozza, F. Brilli, S. Pignattelli, F. Vurro, N. Coppede, M. Bettelli, D. Calestani, F. Loreto, A. Zappettini, Real-time monitoring of *Arundo donax* response to saline stress through the application of in vivo sensing technology. *Sci. Rep.* **11**, 18598 (2021).

11. Q. Xu, S.-Y. Liu, Q.-J. Zou, X.-L. Guo, X.-Y. Dong, P.-W. Li, D.-Y. Song, H. Chen, Y.-D. Zhao, Microsensor in vivo monitoring of oxidative burst in oilseed rape (*Brassica napus* L.) leaves infected by *Sclerotinia sclerotiorum*. *Anal. Chim. Acta* **632**, 21–25 (2009).

12. Q. Xu, F. Wei, Z. Wang, Q. Yang, Y.-D. Zhao, H. Chen, In vivo monitoring of oxidative burst induced by ultraviolet A and C stress for oilseed rape by microbiosensor. *Sens. Actuators B Chem.* **141**, 599–603 (2009).

13. S. Oren, H. Ceylan, P. S. Schnable, L. Dong, High-resolution patterning and transferring of graphene-based nanomaterials onto tape toward roll-to-roll production of tape-based wearable sensors. *Adv. Mater. Technol.* **2**, 1700223 (2017).

14. J. J. Kim, R. Fan, L. K. Allison, T. L. Andrew, On-site identification of ozone damage in fruiting plants using vapor-deposited conducting polymer tattoos. *Sci. Adv.* **6**, eabc3296 (2020).

15. H. H. Felle, The apoplastic pH of the Zea mays root cortex as measured with pH-sensitive microelectrodes: Aspects of regulation. *J. Exp. Bot.* **49**, 987–995 (1998).

16. E. S. McLamore, A. Diggs, P. Calvo Marzal, J. Shi, J. J. Blakeslee, W. A. Peer, A. S. Murphy, D. M. Porterfield, Non-invasive quantification of endogenous root auxin transport using an integrated flux microsensor technique. *Plant J.* **63**, 1004–1016 (2010).

17. N. De Diego, T. Fürst, J. F. Humplík, L. Ugena, K. Podlešáková, L. Spíchal, An automated method for high-throughput screening of *Arabidopsis* rosette growth in multi-well plates and its validation in stress conditions. *Front. Plant Sci.* **8**, 1702 (2017).

18. T. Burrell, S. Fozard, G. H. Holroyd, A. P. French, M. P. Pound, C. J. Bigley, C. James Taylor, B. G. Forde, The Microphenotron: A robotic miniaturized plant phenotyping platform with diverse applications in chemical biology. *Plant Methods* **13**, 10 (2017).

19. Z. Yao, P. Coatsworth, X. Shi, J. Zhi, L. Hu, R. Yan, F. Güder, H.-D. Yu, Paper-based sensors for diagnostics, human activity monitoring, food safety and environmental detection. *Sens. Diagnostics* **1**, 312–342 (2022).

20. H. Y. Y. Nyein, W. Gao, Z. Shahpar, S. Emaminejad, S. Challa, K. Chen, H. M. Fahad, L.-C. Tai, H. Ota, R. W. Davis, A. Javey, A wearable electrochemical platform for noninvasive simultaneous monitoring of Ca^{2+} and pH. *ACS Nano* **10**, 7216–7224 (2016).

21. R. Rahimi, M. Ochoa, T. Parupudi, X. Zhao, I. K. Yazdi, M. R. Dokmeci, A. Tamayol, A. Khademhosseini, B. Ziaie, A low-cost flexible pH sensor array for wound assessment. *Sens. Actuators B Chem.* **229**, 609–617 (2016).

22. W. Zhang, D. Ma, J. Du, Prussian blue nanoparticles as peroxidase mimetics for sensitive colorimetric detection of hydrogen peroxide and glucose. *Talanta* **120**, 362–367 (2014).

23. R. Jiménez-Pérez, J. González-Rodríguez, M. I. González-Sánchez, B. Gómez-Monedero, E. Valero, Highly sensitive H_2O_2 sensor based on poly(azure A)-platinum nanoparticles deposited on activated screen printed carbon electrodes. *Sens. Actuators B Chem.* **298**, 126878 (2019).

24. Z. A. Naveed, X. Wei, J. Chen, H. Mubeen, G. S. Ali, The PTI to ETI continuum in *Phytophthora*-plant interactions. *Front. Plant Sci.* **11**, 593905 (2020).

25. R. Munns, Genes and salt tolerance: Bringing them together. *New Phytol.* **167**, 645–663 (2005).

26. A. v. Barker, D. J. Pilbeam, *Handbook of Plant Nutrition* (CRC Press, ed. 2, 2015).

27. N. M. Crawford, Nitrate: Nutrient and signal for plant growth. *Plant Cell* **7**, 859–868 (1995).

28. C. Dunand, M. Crèvecoeur, C. Penel, Distribution of superoxide and hydrogen peroxide in *Arabidopsis* root and their influence on root development: Possible interaction with peroxidases. *New Phytol.* **174**, 332–341 (2007).

29. M. A. Hossain, S. Bhattacharjee, S.-M. Armin, P. Qian, W. Xin, H. Y. Li, D. J. Burritt, M. Fujita, L.-S. P. Tran, Hydrogen peroxide priming modulates abiotic oxidative stress tolerance: Insights from ROS detoxification and scavenging. *Front. Plant Sci.* **6**, 420 (2015).

30. Y. Y. Chao, Y. T. Hsu, C. H. Kao, Involvement of glutathione in heat shock- and hydrogen peroxide-induced cadmium tolerance of rice (*Oryza sativa* L.) seedlings. *Plant Soil* **318**, 37–45 (2009).

31. A. D. D. A. Neto, J. T. Prisco, J. Enéas-Filho, J. V. Rolim Medeiros, E. Gomes-Filho, Hydrogen peroxide pre-treatment induces salt-stress acclimation in maize plants. *J. Plant Physiol.* **162**, 1114–1122 (2005).

32. Y. Wang, J. Li, J. Wang, Z. Li, Exogenous H_2O_2 improves the chilling tolerance of manilagrass and mascarenegrass by activating the antioxidative system. *Plant Growth Regul.* **61**, 195–204 (2010).

33. M. Ali, Z. Cheng, H. Ahmad, S. Hayat, Reactive oxygen species (ROS) as defenses against a broad range of plant fungal infections and case study on ROS employed by crops against *Verticillium dahliae* wilts. *J. Plant Interact.* **13**, 353–363 (2018).

34. M. A. Torres, J. D. G. Jones, J. L. Dangl, Reactive oxygen species signaling in response to pathogens. *Plant Physiol.* **141**, 373–378 (2006).

35. C. Lamb, R. A. Dixon, The oxidative burst in plant disease resistance. *Annu. Rev. Plant. Physiol. Plant. Mol. Biol.* **48**, 251–275 (1997).

36. B. J. Koo, D. C. Adriano, N. S. Bolan, C. D. Barton, Root exudates and microorganisms. *Encycl Soils Environ*, **4**, 421–428 (2005).

37. R. Crang, S. Lyons-Sobaski, R. Wise, *Plant Anatomy: A Concept-Based Approach to the Structure of Seed Plants* (Springer International Publishing, ed. 1, 2018.

38. R. Gupta, S. K. Chakrabarty, Gibberellic acid in plant. *Plant Signal. Behav.* **8**, e25504 (2013).

39. G. Tyler, A. Zohlen, Plant seeds as mineral nutrient resource for seedlings—A comparison of plants from calcareous and silicate soils. *Ann. Bot.* **81**, 455–459 (1998).

40. H. Zhang, X.-L. Liu, R.-X. Zhang, H.-Y. Yuan, M.-M. Wang, H.-Y. Yang, H.-Y. Ma, D. Liu, C.-J. Jiang, Z.-W. Liang, Root damage under alkaline stress is associated with reactive oxygen species accumulation in rice (*Oryza sativa* L.). *Front. Plant Sci.* **8**, 1580 (2017).

41. S. Fang, X. Hou, X. Liang, Response mechanisms of plants under saline-alkali stress. *Front. Plant Sci.* **12**, 667458 (2021).

42. M. Griffiths, L. M. York, Targeting root ion uptake kinetics to increase plant productivity and nutrient use efficiency. *Plant Physiol.* **182**, 1854–1868 (2020).

43. R. J. Haynes, Active ion uptake and maintenance of cation-anion balance: A critical examination of their role in regulating rhizosphere pH. *Plant Soil* **126**, 247–264 (1990).

44. H. Bülter, F. Peters, J. Schwenzel, G. Wittstock, In situ quantification of the swelling of graphite composite electrodes by scanning electrochemical microscopy. *J. Electrochem. Soc.* **163**, A27–A34 (2016).

45. A. S. P. Collins, H. Kurt, C. Duggan, Y. Cotur, P. Coatsworth, A. Naik, M. Kaisti, T. O. Bozkurt, F. Guder, Parallel, Continuous monitoring and quantification of programmed cell death in plant tissue. bioRxiv 554256 [Preprint]. (2023). https://doi.org/10.1101/2023.08.22.554256.

46. N. Hatsugai, F. Katagiri, Quantification of plant cell death by electrolyte leakage assay. *Bio Protoc.* **8**, e2758 (2018).

47. J. Barros, H. Serk, I. Granlund, E. Pesquet, The cell biology of lignification in higher plants. *Ann. Bot.* **115**, 1053–1074 (2015).

48. L. F. Ruiz Herrera, M. W. Shane, J. López-Bucio, Nutritional regulation of root development. *Wiley Interdiscip. Rev. Dev. Biol.* **4**, 431–443 (2015).

49. S. Penfield, D. R. MacGregor, Effects of environmental variation during seed production on seed dormancy and germination. *J. Exp. Bot.* **68**, 819–825 (2017).

50. P. Mäser, M. Gierth, J. I. Schroeder, Molecular mechanisms of potassium and sodium uptake in plants. *Plant Soil* **247**, 43–54 (2002).

51. A. Wakeel, Potassium–sodium interactions in soil and plant under saline-sodic conditions. *J. Plant Nutr. Soil Sci.* **176**, 344–354 (2013).

52. N. von Wirén, A. Gojon, S. Chaillou, D. Raper, Mechanisms and regulation of ammonium uptake in higher plants, in *Plant Nitrogen* (Springer, 2001), pp. 61–77.

53. V. Demidchik, S. Shabala, S. Isayenkov, T. A. Cuin, I. Pottosin, Calcium transport across plant membranes: Mechanisms and functions. *New Phytol.* **220**, 49–69 (2018).

54. D. Mao, J. Chen, L. Tian, Z. Liu, L. Yang, R. Tang, J. Li, C. Lu, Y. Yang, J. Shi, L. Chen, D. Li, S. Luan, *Arabidopsis* transporter MGT6 mediates magnesium uptake and is required for growth under magnesium limitation. *Plant Cell* **26**, 2234–2248 (2014).

55. N. Arif, V. Yadav, S. Singh, S. Singh, P. Ahmad, R. K. Mishra, S. Sharma, D. K. Tripathi, N. K. Dubey, D. K. Chauhan, Influence of high and low levels of plant-beneficial heavy metal ions on plant growth and development. *Front. Environ. Sci.* **4**, 69 (2016).

56. A. Yan, Y. Wang, S. N. Tan, M. L. Mohd Yusof, S. Ghosh, Z. Chen, Phytoremediation: A promising approach for revegetation of heavy metal-polluted land. *Front. Plant Sci.* **11**, 359 (2020).

57. O. Pettersson, Heavy-metal ion uptake by plants from nutrient solutions with metal ion, plant species and growth period variations. *Plant Soil* **45**, 445–459 (1976).

58. H. Takahashi, Sulfate transport systems in plants: Functional diversity and molecular mechanisms underlying regulatory coordination. *J. Exp. Bot.* **70**, 4075–4087 (2019).

59. J. A. Franklin, J. J. Zwiazek, Ion uptake in *Pinus banksiana* treated with sodium chloride and sodium sulphate. *Physiol. Plant.* **120**, 482–490 (2004).

60. R. Tischner, Nitrate uptake and reduction in higher and lower plants. *Plant Cell Environ.* **23**, 1005–1024 (2000).

61. M. Bucher, Functional biology of plant phosphate uptake at root and mycorrhiza interfaces. *New Phytol.* **173**, 11–26 (2007).

62. J. M. Colmenero-Flores, J. D. Franco-Navarro, P. Cubero-Font, P. Peinado-Torrubia, M. A. Rosales, Chloride as a beneficial macronutrient in higher plants: New roles and regulation. *Int. J. Mol. Sci.* **20**, 4686 (2019).

63. G. P. Ruwanpathirana, D. C. Plett, R. C. Williams, C. E. Davey, L. A. Johnston, H. J. Kronzucker, Continuous monitoring of plant sodium transport dynamics using clinical PET. *Plant Methods* **17**, 8 (2021).

64. C. Moyen, K. E. Hammond-Kosack, J. Jones, M. R. Knight, E. Johannes, Systemin triggers an increase of cytoplasmic calcium in tomato mesophyll cells: Ca^{2+} mobilization from intra- and extracellular compartments. *Plant Cell Environ.* **21**, 1101–1111 (1998).

65. S. Nikoloski, P. Murphy, D. Kocev, S. Džeroski, D. P. Wall, Using machine learning to estimate herbage production and nutrient uptake on Irish dairy farms. *J. Dairy Sci.* **102**, 10639–10656 (2019).

66. D. M. Qiao, H. B. Shi, H. B. Pang, X. B. Qi, F. Plauborg, Estimating plant root water uptake using a neural network approach. *Agric Water Manag* **98**, 251–260 (2010).

67. T. Chen, C. Guestrin, XGBoost: A scalable tree boosting system. arXiv:1603.02754 [cs.LG] (2016).

68. F. Pedregosa, G. Varoquaux, A. Gramfort, V. Michel, B. Thirion, O. Grisel, M. Blondel, P. Prettenhofer, R. Weiss, V. Dubourg, J. Vanderplas, A. Passos, D. Cournapeau, M. Brucher, M. Perrot, É. Duchesnay, Scikit-learn: Machine Learning in Python. *J. Mach. Learn. Res.* **12**, 2825–2830 (2011).

69. J. R. Rumble, Ed., *CRC Handbook of Chemistry and Physics, 103rd Edition* (CRC Press, 2022).

70. S.-H. Lin, H.-F. Kuo, G. Canivenc, C.-S. Lin, M. Lepetit, P.-K. Hsu, P. Tillard, H.-L. Lin, Y.-Y. Wang, C.-B. Tsai, A. Gojon, Y.-F. Tsay, Mutation of the *Arabidopsis* NRT1.5 nitrate transporter causes defective root-to-shoot nitrate transport. *Plant Cell* **20**, 2514–2528 (2008).

71. S. Hanstein, D. de Beer, H. H. Felle, Miniaturised carbon dioxide sensor designed for measurements within plant leaves. *Sens. Actuators B Chem.* **81**, 107–114 (2001).

72. H. M. Rawson, J. E. Begg, R. G. Woodward, The effect of atmospheric humidity on photosynthesis, transpiration and water use efficiency of leaves of several plant species. *Planta* **134**, 5–10 (1977).

73. D. Maier, E. Laubender, A. Basavanna, S. Schumann, F. Güder, G. A. Urban, C. Dincer, Toward continuous monitoring of breath biochemistry: A paper-based wearable sensor for real-time hydrogen peroxide measurement in simulated breath. *ACS Sens.* **4**, 2945–2951 (2019).

74. A. Bukhamsin, A. Ait Lahcen, J. D. O. Filho, S. Shetty, I. Blilou, J. Kosel, K. N. Salama, Minimally-invasive, real-time, non-destructive, species-independent phytohormone biosensor for precision farming. *Biosens. Bioelectron.* **214**, 114515 (2022).

75. V. Krivitsky, E. Granot, Y. Avidor, E. Borberg, R. T. Voegele, F. Patolsky, Rapid collection and aptamer-based sensitive electrochemical detection of soybean rust fungi airborne urediniospores. *ACS Sens.* **6**, 1187–1198 (2021).

76. M. A. Morales, J. M. Halpern, Guide to selecting a biorecognition element for biosensors. *Bioconjug. Chem.* **29**, 3231–3239 (2018).

77. P. Trivedi, J. E. Leach, S. G. Tringe, T. Sa, B. K. Singh, Plant–microbiome interactions: From community assembly to plant health. *Nat. Rev. Microbiol.* **18**, 607–621 (2020).

78. R. T. Furbank, M. Tester, Phenomics—Technologies to relieve the phenotyping bottleneck. *Trends Plant Sci.* **16**, 635–644 (2011).

79. C. S. Widodo, H. Sela, D. R. Santosa, The effect of NaCl concentration on the ionic NaCl solutions electrical impedance value using electrochemical impedance spectroscopy methods. *AIP Conf. Proc.* **2021**, 050003 (2018).

80. L. F. Lima, A. L. Vieira, H. Mukai, C. M. G. Andrade, P. R. G. Fernandes, Electric impedance of aqueous KCl and NaCl solutions: Salt concentration dependence on components of the equivalent electric circuit. *J. Mol. Liq.* **241**, 530–539 (2017).

81. Y. Liu, D. M. Li, J. Qian, B. Di, G. Zhang, Z. H. Ren, Electrical impedance spectroscopy (EIS) in plant roots research: A review. *Plant Methods* **17**, 118 (2021).

82. I. Jócsák, G. Végvári, E. Vozáry, Electrical impedance measurement on plants: A review with some insights to other fields. *Theor. Exp. Plant Physiol.* **31**, 359–375 (2019).

83. W. J. Frierson, M. J. Ammons, The separation of inorganic ions by paper partition chromatography. *J. Chem. Educ.* **27**, 37–38 (1950).

84. T. Lindfors, A. Ivaska, pH sensitivity of polyaniline and its substituted derivatives. *J. Electroanal. Chem.* **531**, 43–52 (2002).

85. G. D. M. Madeira, H. J. Hugo, M. C. Faleiros, M. Mulato, Model improvement for super-Nernstian pH sensors: The effect of surface hydration. *J. Mater. Sci.* **56**, 2738–2747 (2021).

86. M. I. González-Sánchez, L. González-Macia, M. T. Pérez-Prior, E. Valero, J. Hancock, A. J. Killard, Electrochemical detection of extracellular hydrogen peroxide in *Arabidopsis thaliana*: A real-time marker of oxidative stress. *Plant Cell Environ.* **36**, 869–878 (2013).
